# T follicular regulatory cells in food allergy promote IgE via IL-4

**DOI:** 10.1172/jci.insight.171241

**Published:** 2024-10-08

**Authors:** Qiang Chen, Abdullahi M. Abdi, Wei Luo, Xue Yuan, Alexander L. Dent

**Affiliations:** 1 Department of Microbiology and Immunology and; 2 Department of Otolaryngology – Head and Neck Surgery, Indiana University School of Medicine, Indianapolis, Indiana, USA.

**Keywords:** Immunology, Allergy, Immunoglobulins, T cells

## Abstract

T follicular regulatory (TFR) cells are found in the germinal center (GC) response and, along with T follicular helper (TFH) cells, help to control the development of high-affinity antibodies (Ab). Although TFR cells are generally thought to repress GC B cells and the Ab response, we have previously shown that in a mouse food allergy model, TFR cells produce IL-10 and play an essential helper role such that in the absence of TFR cells, IgE responses are diminished. Here we show that in this food allergy response, TFR cells produced IL-4 that promotes the generation of antigen-specific IgE. We show that food allergy–primed TFR cells specifically upregulate IL-4 gene transcription and produce functional IL-4 that promoted IgE responses both in vitro and in vivo. We determined that IgE responses are dependent on a high level of IL-4 produced by follicular T cells in the GC, explaining the need for IL-4 produced by TFR cells in the food allergy response. Overall, our findings have demonstrated that in food allergy, TFR cells can produce IL-4 and regulate IgE in a manner that augments the role of TFH cells in IgE responses.

## Abstract 2

In food allergy, TFR cells can produce IL-4 and regulate IgE in a manner that is complementary to the role of TFH cells in IgE responses.

## Graphical abstract

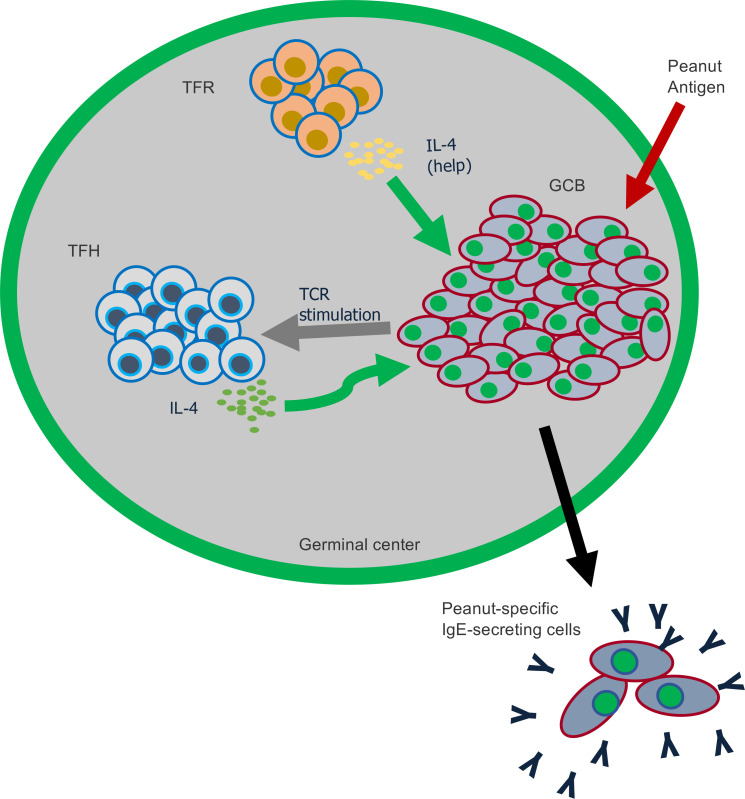

## Introduction

The prevalence of food allergy has increased in the past few decades as part of the overall epidemic growth in allergic disease (1, 2). Up to 8% of children are affected by at least 1 type of food allergy in the United States (3). Food allergy occurs due to an abnormal immune response to food, where Immunoglobin E (IgE) antibodies (Abs) develop that are specific to food antigens (Ags). Food-specific IgE can then activate the allergic response upon reexposure to the food Ag. How IgE responses develop in food allergy is not fully understood.

IgE is regulated by several cytokines, and the most important among them is IL-4. IL-4 promotes class switching to both IgE and IgG1 by activating STAT6 (4–6). However, stimulation of mouse B cells with CD40 signaling in the presence of IL-4 induces more class switching to IgG1 than IgE (7, 8). This may reflect that the switch region of the IgE gene is much shorter than that of IgG1, making it a less frequent target for activation-induced cytidine deaminase (AID), the key enzyme that mediates class switching (9). Consistent with IgE being a low frequency target for class switching, Ag-specific IgE but not IgG1 is impaired in mice heterozygous for the *Il4* gene (10), suggesting that switching to IgE is more sensitive to the level of IL-4 than switching to IgG1 (9). However, another study demonstrated that early Iε transcription, which occurs prior to switching to IgE, occurs at a lower activation level than for IgG1 (11). However, the differential regulation of allergen-specific IgE and IgG1 responses in the food allergy model has not been analyzed in detail.

Though the original paradigm was that Th2 cells promote IgE responses because they produce IL-4, recent papers have shown that IgE development and IgE-mediated allergic responses are dependent on T follicular helper (TFH) cells and TFH cell–derived IL-4 (12–17). Another cell population, called the T follicular regulatory (TFR) cell, has been identified as playing an essential role in regulating the food allergy IgE response (18). TFR cells are typically derived from Foxp3^+^ Tregs that have upregulated the key TFH cell transcription factor Bcl6 such that TFR cells have a hybrid Treg/TFH phenotype (19–21). Strikingly, while TFR cells promote the IgE response in a mouse food allergy model, TFR cells limit the IgE response in a mouse airway inflammatory asthma model (18, 22, 23). The regulatory pathways that control whether TFR cells act as helper or suppressor cells are not understood. At least part of the helper role of TFR in IgE response in the food allergy system is mediated by IL-10 (18). In traditional protein-adjuvant immunization models, the suppressor factors CTLA4, Neuritin, and FGL2 expressed by TFR cells were shown to inhibit the IgE response (24–26). Whether TFR cell–derived IL-10 explains the helper role of TFR cells in food allergy or whether there are other mechanisms used by TFR cells to promote the IgE response in food allergy is unclear. One specific possibility that has not been addressed is whether, along with IL-4 produced by TFH cells (27–29), TFR cells produce IL-4 that contributes to IgE switching. Noval Rivas et al. show that Treg-derived IL-4 was required for the IgE response in a mouse food allergy model (30), but they did not examine TFR cells. Here, we decided to test whether TFR cells produce meaningful levels of IL-4 and, if so, whether TFR-derived IL-4 can regulate the IgE response in our food allergy model.

In this study, we found that, in a mouse food allergy model, TFR cells produce levels of IL-4 near the levels of IL-4 produced by TFH cells. We further show that, in this model, the IL-4 produced by TFR cells contributes to the development of Ag-specific IgE but not IgG1. Our results additionally indicate that the levels of IL-4 and IL-4–producing follicular T cells are both critical for IgE responses, providing an explanation for why TFR cell–derived IL-4 may be required for the development of IgE in food allergy.

## Results

### Comparable production of IL-4 by both TFR and TFH cells.

**Figure 1:**
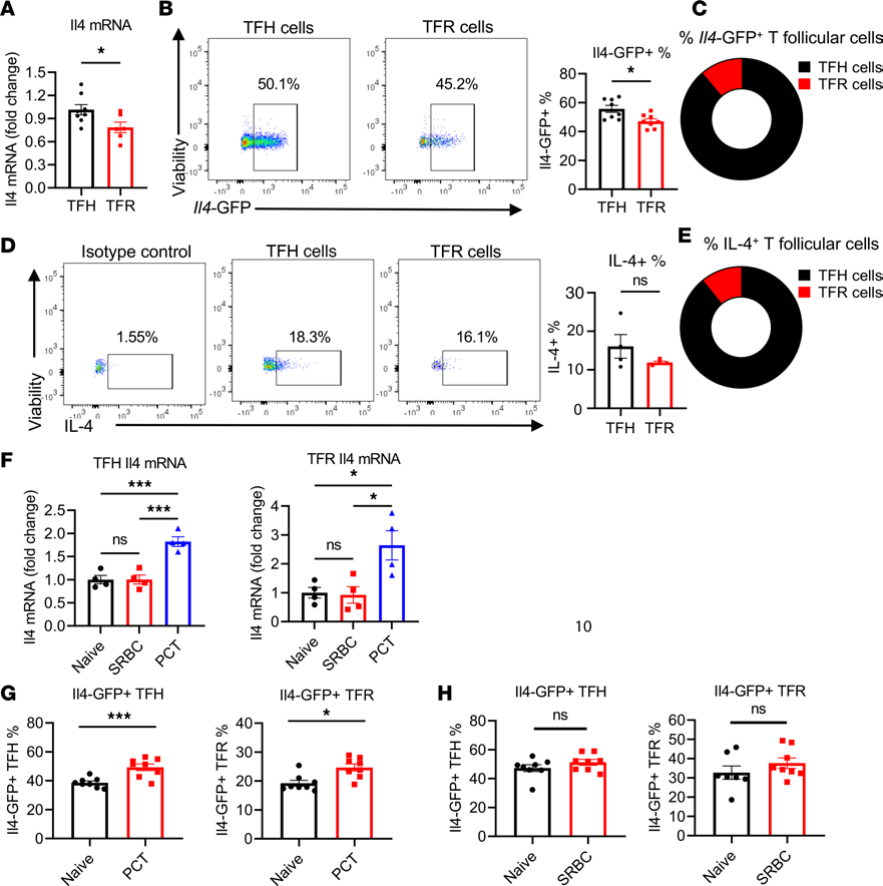
Comparable expression of *Il4* gene between TFR and TFH cells. (**A**) *Il4* mRNA expression in TFH and TFR cells. Foxp3-YFP mice were sensitized with PCT at day 1 and day 8; then, TFH and TFR cells were sorted from mesenteric lymph nodes (mLN) at day 12 (Supplemental Figure 1A). *Il4* mRNA expression measured using qPCR. Data were combined from 2 independent experiments. *n* = 6–8. (**B**) *Il4*-GFP^+^ TFH and TFR cells in 4Get IL-4–reporter mice. Cells from mLN were analyzed using flow cytometry at day 12 after PCT sensitizations. *n* = 8. (**C**) Pie chart of average percentage of TFH and TFR cells within the total *Il4*-GFP^+^ follicular T cell population. Cells were gated first on CD4^+^CXCR5^+^PD-1^hi^IL4-GFP^+^ and then gated for Foxp3^+^ (TFR) and Foxp3^–^ (TFH) cells. (**D**) IL-4–expressing TFH and TFR cells as in **A** analyzed by intracellular cytokine staining. *n* = 4. (**E**) Pie chart of average percentage of TFH and TFR cells within the IL-4–expressing follicular T cells in (**D**). Cells were gated first on CD4^+^CXCR5^+^PD-1^hi^IL-4^+^ and then gated for Foxp3^+^ (TFR) and Foxp3^–^ (TFH) cells. (**F**) *Il4* mRNA expression in TFH and TFR cells from naive mice and mice with SRBC immunization or PCT sensitization. TFH and TFR cells were sorted from mLN plus spleen. *n* = 4. (**G**) *Il4*-GFP^+^ TFH and TFR cells from naive mice versus mice sensitization as in **A**, analyzed by flow cytometry. *n* = 8. (**H**) *Il4*-GFP^+^ TFH and TFR cells from naive mice and SRBC immunized mice at day 15. Spleen cells were analyzed by flow cytometry. *n* = 8. *P < 0.05, ***P < 0.001 by *t* test (**A**–**D**, **G**, and **H**) or 1-way ANOVA (**F**). Data are representative of 2 independent experiments.

A helper role for TFR cells in food allergy was previously demonstrated with the use of TFR cell–deficient *Foxp3*^Cre^*Bcl6*^fl/fl^ (Bcl6FC) mice (18). In this food allergy model, peanut protein plus cholera toxin (PCT) is administered intragastrically (i.g.), leading to the production of high levels of peanut-specific (PN-specific) IgE that can promote anaphylaxis following injection of peanut protein (18). The production of IgE in this model requires at least 2 PCT exposures, and the timing of the initial 2 sensitization steps is critical for IgE production (31). Given the complexity of IgE induction in this model, we wondered if TFR cell factors besides IL-10 might contribute to the IgE response. Based on the Noval Rivas et al. (30) findings of Treg-derived IL-4 being important for IgE production in food allergy and RNA-Seq data from our earlier study showing that food allergy–induced TFR cells express *Il4* mRNA (18), we specifically wondered if TFR cells produced IL-4 that could contribute to the IgE response. To explore this possibility, we examined IL-4 production by TFR cells in greater detail. We therefore performed PCT food allergy sensitization in mice and then isolated TFH and TFR cells (Supplemental Figure 1A; supplemental material available online with this article; https://doi.org/10.1172/jci.insight.171241DS1) and examined *Il4* mRNA expression. Quantitative PCR (qPCR) analysis showed that, in PCT sensitized mice, TFR cells express substantial levels of *Il4* mRNA, ~75% of the amount of *Il4* mRNA produced by TFH cells (Figure 1A). To further assess *Il4* expression by TFR cells, we used *Il4* reporter mice, where cells activated to transcribe *Il4* also express enhanced GFP (EGFP) (32). After PCT sensitization, flow cytometry for GFP^+^ cells showed that the average percentage of *Il4*-GFP^+^ cells within the total TFR population (46%) is close to but still significantly less than the average percentage of *Il4*-GFP^+^ cells within the total TFH population (56%) (Figure 1B). At the same time, the absolute number of IL-4–expressing TFR cells is 12% of the absolute number of IL-4–expressing TFH cells (Supplemental Figure 1B). Similarly, among *Il4*-GFP^+^ T follicular cells, 11% of them are TFR cells (Figure 1C). These data indicate that IL-4–expressing TFR cells are a relatively small but substantial population of IL-4–expressing follicular T cells.

We then assessed if TFR cells were the only Foxp3^+^ Treg-related cells expressing IL-4 in this model. Using CXCR5 and PD-1 expression, CD4^+^Foxp3^+^ Tregs can be divided into 4 populations: (a) CXCR5^–^PD-1^–^, (b) CXCR5^+^PD-1^–^ (pre-TFR), (c) CXCR5^–^PD-1^+^, and (4) CXCR5^hi^PD-1^hi^ (TFR) cells (Supplemental Figure 2A). We found that, of these 4 subpopulations, only the pre-TFR and TFR subpopulations express *Il4* mRNA (Supplemental Figure 2B), indicating that *Il4* mRNA expression is induced in these cells as they transition to the TFR stage. However, the TFR subpopulation expressed significantly more *Il4* mRNA than the pre-TFR subpopulation (Supplemental Figure 2A).

To test actual IL-4 protein expression by TFR cells, we isolated CD4^+^ T cells from PCT sensitized mice for intracellular cytokine staining. A similar percentage of IL-4–expressing TFR cells was observed as compared with TFH cells (Figure 1D). By this method, ~12% of IL-4–expressing T follicular cells were TFR cells (Figure 1E). In summary, we found that TFR cells produce nearly comparable levels of IL-4 as TFH cells in this peanut-based food allergy model.

Next, we tested whether TFR cells produce IL-4 specifically in the food allergy model. We measured *Il4* mRNA in TFH and TFR cells from naive mice, mice immunized with sheep RBCs (SRBC), and mice sensitized with PCT. qPCR analysis shows that both TFH and TFR cells had significantly higher expression of *Il4* mRNA after PCT sensitizations compared with naive mice and mice immunized with SRBC (Figure 1F). This increase of *Il4* mRNA in TFH and TFR cells after PCT sensitizations was further validated using *Il4* reporter (4Get) mice (Figure 1, G and H). The high *Il4*-GFP signal in naive TFH and TFR cells likely reflects open chromatin at the *Il4* gene locus and does not necessarily show the percentage of cells expressing IL-4 protein (33). Nonetheless these data still reveal a higher capacity for IL-4 production in the PCT primed TFR cells. Strikingly, in contrast to CXCR5^hi^PD-1^hi^ TFR cells, CXCR5^+^PD-1^–^ pre-TFR cells did not show increased *Il4*-GFP expression after PCT sensitization (Supplemental Figure 2B), indicating that the increased *Il4* expression in TFR cells after PCT treatment occurred during or after the transition from the pre-TFR to TFR stage. To further characterize the timing of expression of *Il4* expression in TFR cells, we examined the *Il4* mRNA at day 8 after 1 sensitization and day 12 after 2 sensitizations. While TFH cells showed statistically significant upregulation of *Il4*-GFP expression after a second PCT sensitization, TFR cells did not show statistically significant *Il4*-GFP upregulation after the second PCT sensitization (Supplemental Figure 2C). Since the high basal expression of *Il4-*GFP in TFR cells in naive mesenteric lymph nodes (mLN) was striking (Figure 1G), we also examined other peripheral LNs and spleen in naive mice. We observed that *Il4*-GFP^+^ TFR cell levels in mLNs were significantly higher than other peripheral LNs and the spleen in naive mice (Supplemental Figure 2D), indicating that the gut environment is poised for higher IL-4 gene expression.

Overall, we found that TFR cells strongly upregulate IL-4 specifically after food allergy sensitization but that the gut environment may be especially primed for induction of IL-4 in TFR cells. This pattern of IL-4 gene expression in TFR cells was also seen by Georgiev et al. in their analysis of Peyer’s patch versus peripheral LN TFR cells (34).

### Regulation of Il4 expression in TFR cells in food allergy.

**Figure 2:**
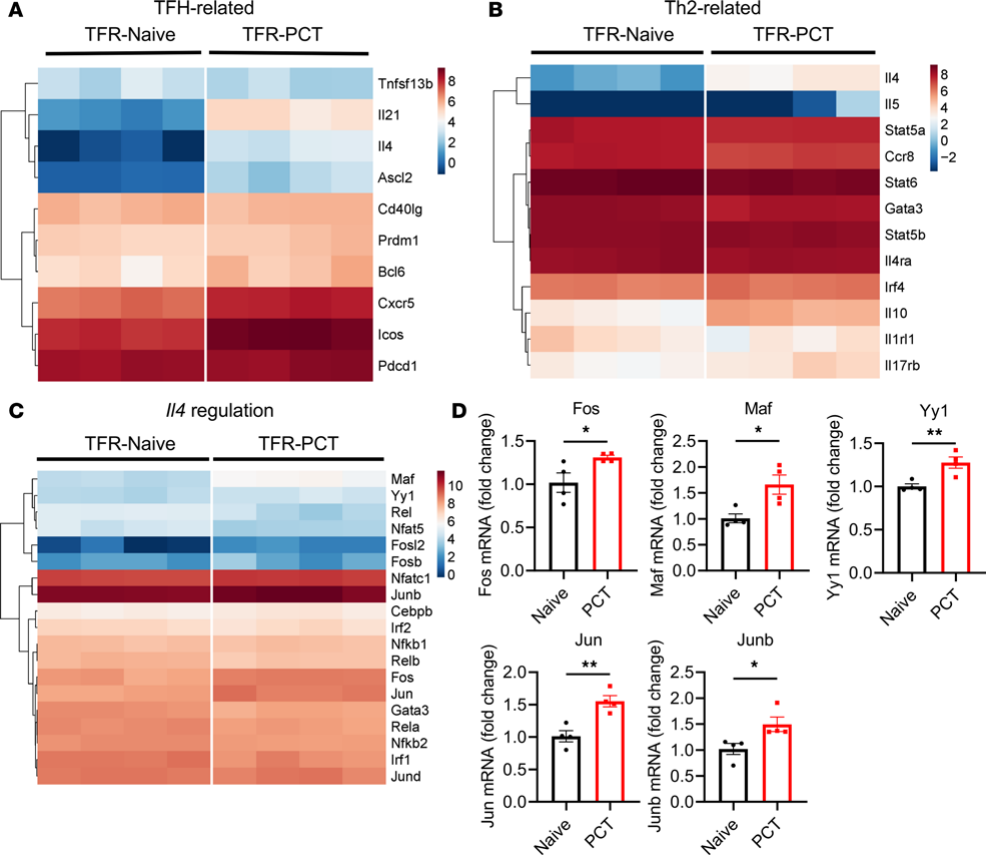
Gene profiling of naive TFR cells and TFR cells in food allergy. (**A**–**C**) Heatmap of selected genes in TFR cells. TFR cells were sorted from the mLNs plus spleen of naive mice and mice at day 12 after PCT sensitization at day 1 and day 8. Total RNA was extracted and subjected to bulk RNA-Seq and analysis. Genes associated with TFH cells (**A**), Th2 cells (**B**), and *Il4* gene regulation (**C**) were compared between naive mice and PCT-sensitized mice. The heatmap represents the values of log_2_FPKM. Significance was defined by |log_2_FC| > 1 and adjusted *P* < 0.05. (**D**) mRNA expression of selected genes in TFR cells by qPCR. TFR cells were sorted from naive and PCT-sensitized mice. RNA was extracted, and the first-strand cDNA was synthesized. Levels of mRNA for selected genes was measured using qPCR. *n* = 4. *P < 0.05, **P < 0.01 by *t* test (**D**). Data were from 1 independent experiment. Heatmaps were created using pheatmap package with clustering rows in R (4.3.1).

To further understand the nature of IL-4–expressing TFR cells in our food allergy model, we sorted TFR cells from the mLNs of naive and PCT-sensitized mice and performed bulk RNA-Seq. We observed higher expression of several TFH cell–associated genes in PCT-sensitized TFR cells over naive TFR cells, including *Il4*, *Il21*, *Cxcr5*, *Icos*, and *Ascl2* (Figure 2A). Along with higher *Il4* expression, TFR cells also express higher levels of Th2 genes such as *Il5*, *Il10*, and *Il17rb* after PCT sensitization (Figure 2B). These data indicate some polarization of the TFR cells to the Th2 lineage; however, the Th2 gene *Il13* was not detected in any TFR cells by RNA-Seq, and expression of the Th2 lineage defining factor *Gata3* was slightly lower in PCT-sensitized TFR cells compared with naive TFR cells (Figure 2, B and C). To better understand how *Il4* gene expression is controlled in PCT-sensitized TFR cells, we compared the expression of *Il4* gene regulation–associated transcription factor genes, particularly genes in the AP-1, NFAT, and NF-κB gene families (Figure 2C) (35). The expression of *Fos* and *Maf* is significantly higher in PCT-sensitized TFR cells than in naive TFR cells, whereas several NF-κB gene family members (*Nfkb2, Rel, Rela, Relb*) were expressed lower in the PCT-sensitized TFR cells at different levels (Figure 2, C and D). Significantly increased expression of *Fos*, *Jun*, *Junb*, *Maf*, and *Yy1* in PCT-sensitized TFR cells was validated using qPCR (Figure 2D). To understand whether this pattern of *Il4-*regulating transcription factor expression was unique to TFR cells, we compared expression of these genes between PCT-induced TFH and TFR cells. Notably, the food allergy TFH and TFR cells showed very similar patterns of *Il4-*regulating transcription factor gene expression (Supplemental Figure 3), indicating parallel modes of IL-4 gene regulation. Overall, our data indicate that the increase in *Il4* expression in food allergy primed TFR cells is not due to enhanced Th2 polarization and may be explained by increased activity from AP-1 and Maf transcription factors.

### TFR-derived IL-4 contributes to Ag-specific IgE by adoptive transfer.

Since TFR cells produce substantial levels of IL-4 in the PCT food allergy model, and our previous data show that TFR cells were critical for the production of Ag-specific IgE in this model (18), we hypothesized that TFR cells act as an important source of IL-4 for the IgE response in the PCT food allergy model.

**Figure 3:**
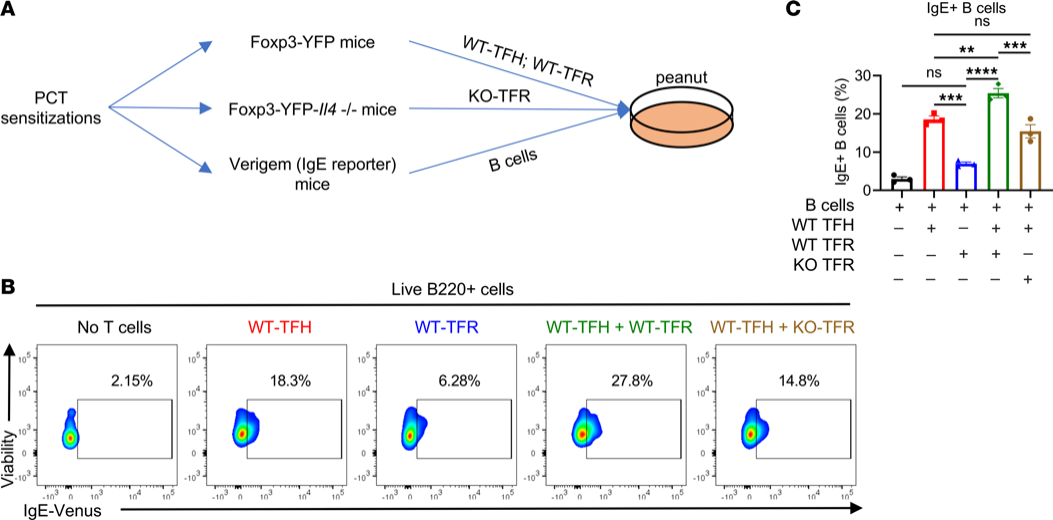
TFR-derived IL-4 promotes antigen-specific IgE in vitro. (**A**) Set-up of the in vitro coculture assay. Foxp3-YFP, Foxp3-YFP-*Il4*^–/–^, and Verigem IgE reporter mice were sensitized with PCT at day 1 and day 8. WT-TFH (CXCR5^+^PD-1^+^YFP^–^), WT-TFR (CXCR5^+^PD-1^+^YFP^+^), *Il4*^–/–^ TFR (CXCR5^+^PD-1^+^YFP^–^, KO-TFR), and B cells (Verigem) were isolated from mice at day 15. They were then cocultured as indicated. Peanut protein extract was added into the culture at 20 μg/mL. Four days later, IgE^+^ B cells were analyzed by flow cytometry. (**B**) Flow cytometric plots of IgE^+^ B cells after 4 days of culture. Color contours show the Venus fluorescence of the Verigem IgE reporter. (**C**) IgE^+^ B cells in coculture stimulated with peanut protein extract. Mice were sensitized with PCT, and cells were isolated for in vitro coculture as indicated. *n* = 3. **P < 0.01, ***P < 0.001, ****P < 0.0001 by 1-way ANOVA (**C**). Data are representative of 2 independent experiments.

To test this idea, we first used an in vitro coculture system to investigate the role of TFR cell–derived IL-4 in regulating the IgE response. We modified an in vitro system used by Clement et al. to test the regulation of IgE by TFR cells in an allergic airway model (36). As diagrammed in Figure 3A, Foxp3-YFP, Foxp3-YFP-*Il4*^–/–^, and Verigem IgE reporter mice were sensitized with PCT; then *Il4-*sufficient WT-TFH and WT-TFR cells were sorted from Foxp3-YFP (WT) mice, *Il4-*deficient KO-TFR cells were sorted from Foxp3-YFP-*Il4*^–/–^, and B cells were sorted from Verigem mice. Different combinations of TFH and TFR cells were then cocultured with Verigem B cells and peanut Ag for 4 days. After 4 days, IgE^+^ B cells were analyzed by flow cytometry. TFH cells alone induced a substantial IgE response in the B cells, whereas TFR cells alone induced a slight increase that did not reach statistical significance (Figure 3, B and C). However, addition of WT-TFR cells to the TFH cells significantly increased the percent of IgE^+^ B cells compared with TFH cells alone (Figure 3, B and C). In contrast, addition of KO-TFR cells with the TFH cells induced a comparable or slightly lower level of IgE^+^ B cells, indicating that TFR cell–mediated enhancement of the IgE response in this system is mediated by IL-4 (Figure 3, B and C).

**Figure 4:**
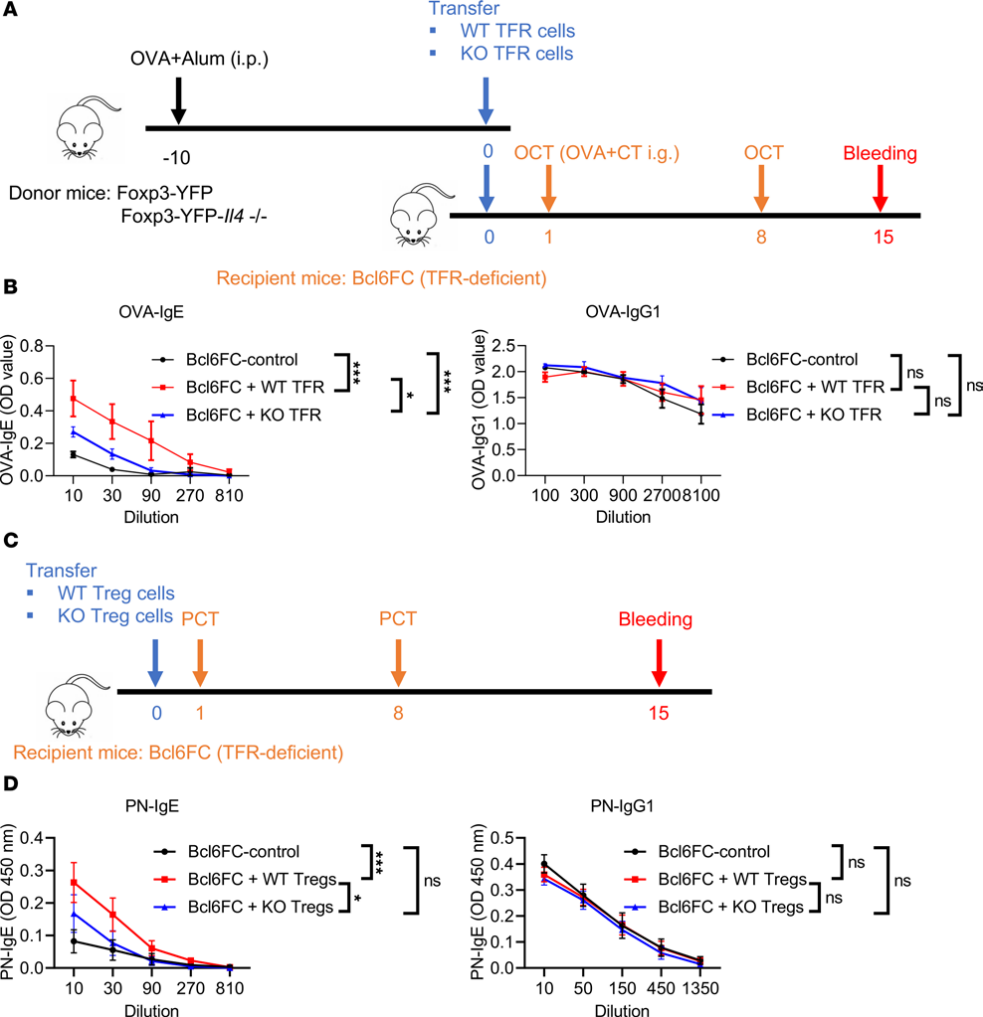
TFR-derived IL-4 contributes to antigen-specific IgE but not IgG1 in vivo. (**A**) Set-up of the TFR cell transfer assay. Foxp3-YFP and Foxp3-YFP-*Il4*^–/–^ mice were immunized with OVA plus Alum 10 days before transfer. WT-TFR cells and KO-TFR cells were sorted and transferred to TFR-deficient Bcl6FC mice. These recipient Bcl6FC mice were sensitized with OVA plus cholera toxin at day 1 and day 8, and then sera were collected at day 15. (**B**) OVA-specific IgE and IgG1 in Bcl6FC mice receiving WT-TFR or KO-TFR cells transfer. Sera were collected after sensitizations, and OVA-specific IgE and IgG1 were tested. *n* = 4–5. (**C**) Set-up of the Treg transfer assay. Tregs were isolated from Foxp3-YFP and Foxp3-YFP-*Il4*^–/–^ mice and then transferred into TFR-deficient Bcl6FC mice. These recipient Bcl6FC mice were sensitized with PCT at day 1 and day 8, and then sera were collected at day 15. (**D**) PN-specific IgE and IgG1 in Bcl6FC mice receiving WT-Treg or KO-Treg transfer. *n* = 10. Sera were collected after sensitizations, and PN-specific IgE and IgG1 were tested. *P < 0.05, ***P < 0.001 by 2-way ANOVA (**B** and **D**). Data were representative of 2 independent experiments (**B**) or combined from 2 independent experiments (**D**).

We next used in vivo cell transfer to study the role of TFR cell–derived IL-4 on IgE responses. As noted earlier, TFR-deficient Bcl6FC mice have a greatly impaired PN-specific IgE response in the food allergy model (18). Therefore, we sought to determine whether the adoptive transfer of WT TFR cells to Bcl6FC mice could rescue the phenotype of Bcl6FC mice. To perform this experiment, we first immunized WT and *Il4*^–/–^ mice with OVA plus Alum (i.p.) in order to obtain a larger number of Ag-specific TFR cells for transfer than could be obtained from mLN. We had previously used OVA plus CT in the food allergy model and observed a similar response as with peanut plus CT (18). After OVA-Alum immunization, we sorted WT-TFR cells and *Il4*^–/–^ TFR (KO-TFR) cells from the TFR donor mice, and we then transferred (i.v.) these types of TFR cells into different groups of recipient Bcl6FC mice (Figure 4A). After OVA plus CT (i.g.) sensitizations, OVA-specific IgE and IgG1 were tested (Figure 4B). Our data show that, compared with Bcl6FC mice without cell transfer, adoptive transfer of WT-TFR cells significantly helped the Bcl6FC mice develop PN-specific IgE (Figure 4B). Consistent with a role for TFR derived IL-4 being critical in the IgE response, adoptive transfer of *Il4*^–/–^ TFR cells was unable to increase OVA-specific IgE as much as WT TFR cells transfer, although the KO-TFR were able to significantly enhance the OVA-specific IgE response (Figure 4B). Strikingly, OVA-specific IgG1 was comparable between these mice (Figure 4B), showing that the effect of TFR cells in this system is primarily on IgE response. We next wondered if transfer of bulk WT Tregs to Bcl6FC mice could also rescue the IgE response, since these cells could develop into TFR cells during the sensitization response. When we transferred total Tregs to Bcl6FC mice, we observed similar results as with the transfer of TFR cells (Figure 4C), where WT Tregs but not *Il4*^–/–^ Tregs rescued the loss of Ag-specific IgE in Bcl6FC mice (Figure 4D). Thus, TFR-derived IL-4 promotes the Ag-specific IgE response in this food allergy model but has little effect on the IgG1 response.

### Ag-specific IgE but not IgG1 is strongly affected by IL-4 availability.

**Figure 5:**
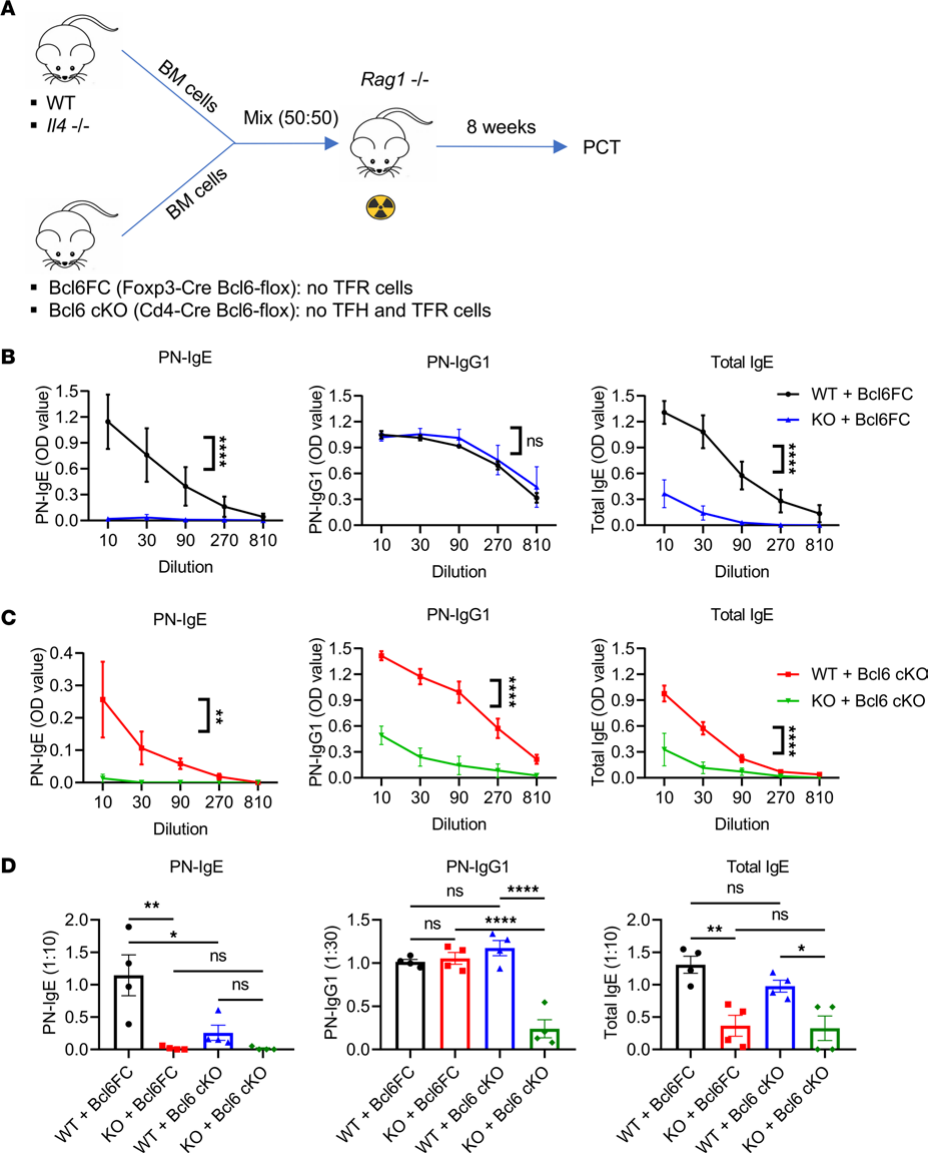
Antigen-specific IgE and IgG1 are differentially affected by IL-4 produced by follicular T cells. (**A**) Set-up of BM chimera (BMC) mice. BM cells from WT or *Il4*^–/–^ mice were mixed 50% + 50% with BM cells from Bcl6FC or Bcl6 cKO mice. Mixed BM cells were injected (i.v.) into irradiated *Rag1*^–/–^ mice. Eight weeks later, BMC mice were sensitized with PCT. (**B**) PN-specific IgE, PN-specific IgG1, and total IgE in Bcl6FC BMC mice sensitized with PCT at day 1 and day 8. Sera were collected, and Abs were tested at day 15. *n* = 4. (**C**) PN-specific IgE, PN-specific IgG1, and total IgE in Bcl6 cKO BMC mice sensitized with PCT at day 1 and day 8. Sera were collected, and Abs were tested at day 15. *n* = 4. (**D**) Levels of PN-specific IgE, PN-specific IgG1, and total IgE for both Bcl6FC and Bcl6 cKO BMC shown at specific titers to allow direct comparison of data from **B** and **C** above. *P < 0.05, **P < 0.01, ****P < 0.0001 by 2-way ANOVA (**B** and **C**) or 1-way ANOVA (**D**). Data were representative of 2 independent experiments.

We next sought to expand on the previous results showing that IL-4–producing TFR cells could enhance IgE responses by using a BM chimera (BMC) system, where we could compare the function of endogenous IL-4–producing TFR cells versus endogenous IL-4 KO-TFR cells on the food allergy IgE response. This was accomplished with the scheme shown in Figure 5A, where Bcl6FC BM was mixed 50:50 with BM from either WT or *Il4*^–/–^ mice and injected into sublethally irradiated Rag1^–/–^ mice. After the BMCs developed an immune system, we primed them for food allergy with PCT. Whereas mice with WT TFR cells (50% WT BM and 50% Bcl6FC BM) produced a robust IgE response (both PN-specific and total IgE), mice with IL-4 KO-TFR cells (50% *Il4*^–/–^ BM and 50% Bcl6FC BM) showed a complete loss of PN-specific IgE and a nearly ablated total IgE response (Figure 5B). As in the TFR transfer experiments, the IgG1 response was not affected by alterations in TFR cells (Figure 5B). While these data support the idea that TFR-derived IL-4 is critical for the IgE response, we could not rule out that in 50% *Il4*^–/–^ plus 50% Bcl6FC BMC mice, 50% of the TFH cells in the chimeric system would develop from *Il4*^–/–^ BM. This would mean that, in these BMC, 50% of the IL-4–producing TFH cells would be lost, which could lead to the effects on IgE we observed. Therefore, in a related set of chimeras, using CD4-Cre Bcl6-flox (Bcl6 cKO) mice instead of Bcl6FC mice, we also tested the broader role of IL-4–producing TFH and TFR cells on the IgE response (Figure 5A). In this set of BMCs, mice in which only WT TFH and TFR cells developed (50% WT BM and 50% Bcl6 cKO BM) produced a significant IgE response (both PN-specific and total IgE) (Figure 5B). In contrast, in mice in which only IL-4 KO-TFH and IL-4 KO-TFR cells developed (50% IL-4 KO BM and 50% Bcl6 cKO BM) showed a complete loss in PN-specific IgE and a nearly ablated total IgE response (Figure 5C). In contrast to the TFR transfer experiments, the IgG1 response was also greatly depleted in mice with IL-4 KO-TFH and IL-4 KO-TFR cells (Figure 5C), clearly showing a role for IL-4–producing TFH cells in the PN-specific IgG1 response. These Bcl6 cKO BMCs revealed a major role for IL-4–producing TFH cells for both PN-specific IgE and IgG1 responses and further suggest that the loss of IgE in the Bcl6FC TFR chimeras was due to the loss of 50% of the IL-4–producing TFH cells.

We then directly compared the Ab response between the 2 types of BMC systems (Figure 5D). This comparison revealed that the PN-specific IgE response was significantly weaker in the BMC with loss of 50% TFH cell production (WT + Bcl6 cKO) versus the BMC that had 50% loss of TFR cells (WT + Bcl6FC). The PN-specific IgG1 and total IgE responses were not significantly affected by loss of 50% TFH cells. Nonetheless, these data suggest that loss of 50% of the TFH and TFR cells — where only 50% of the responding CD4 T cells can develop into TFH and TFR cells — leads to a strongly decreased PN-specific IgE response, even though WT TFH/TFR cells still develop. However, this was not a definitive result because we did not have a full WT BMC control and used 50% WT plus 50% Bcl6FC BMC mice as a control. We therefore tested loss of 50% TFH/TFR cells in a set of BMCs where we could carefully assess the cells that developed (Supplemental Figure 4A). Using 50% WT CD45.1 (BoyJ) BM mixed with 50% either WT CD45.2 (B6) BM or Bcl6 cKO CD45.2 BM, we determined that the BM proportions were correct and that, after food allergy sensitization, roughly ~50% of each type of TFH and TFR cell developed in the WT(BoyJ):WT(B6) BMCs (Supplemental Figure 4, B and C). In the WT:Bcl6 cKO BMCs, the Bcl6 cKO was fully deleting TFH and TFR cells and all TFH and TFR cells were derived from the BoyJ BM (Supplemental Figure 4, B and C). We noted that, in the WT:Bcl6 cKO BMCs, the BoyJ T cells did not fully compensate for loss of 50% of the TFH and TFR cells and only generated the same percentage of T cells as in the WT:WT BMCs (Supplemental Figure 4C). When we tested the PN-specific IgE in these BMCs, we saw a very large decrease in the WT:Bcl6 cKO BMC response compared with the WT:WT BMC response (Supplemental Figure 4D). These data confirm the marked loss of PN-specific IgE in WT:Bcl6 cKO BMC mice in Figure 5. Overall, these data clearly show that loss of 50% of TFH and TFR cells leads to a dramatic loss of the PN-specific IgE response, likely because these 2 cell types are both producing IL-4 that drives the IgE response in the germinal center (GC).

**Figure 6:**
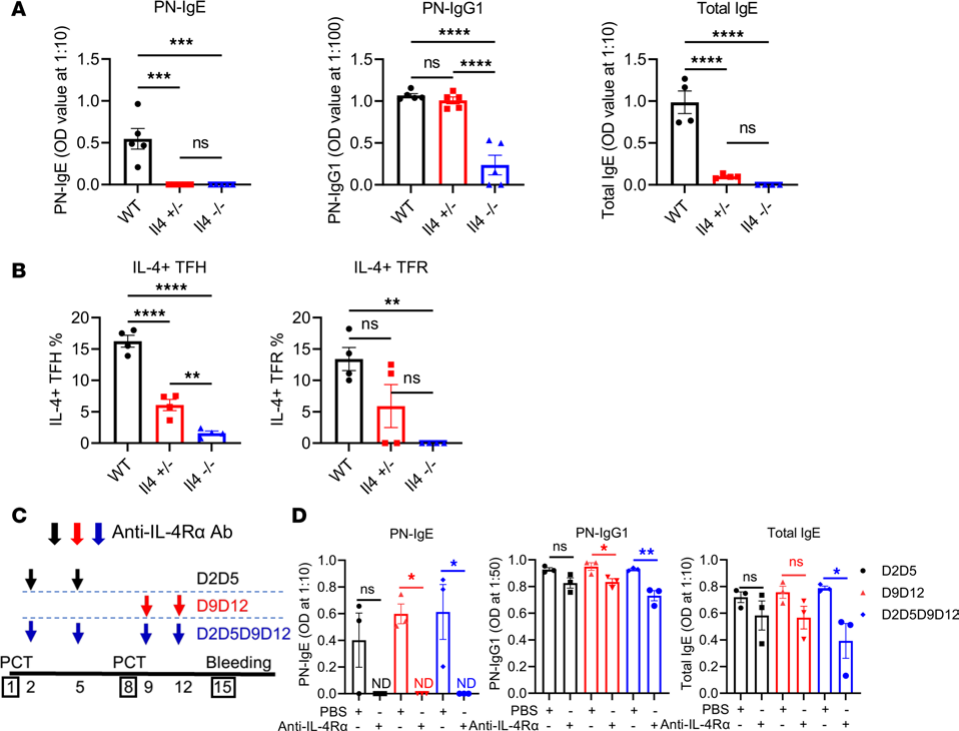
Antigen-specific IgE and IgG1 are differentially affected by IL-4 levels. (**A**) PN-specific IgE, PN-specific IgG1, and total IgE in WT, *Il4*^+/–^, and *Il4*^–/–^ mice after PCT. Mice were sensitized with PCT at day 1 and day 8. Sera were collected at day 15, and Abs were tested using ELISA. *n* = 4. (**B**) IL-4^+^ TFH and TFR cells in WT, *Il4*^+/–^, and *Il4*^–/–^ mice. IL-4–expressing cells were measured by flow cytometry after PCT sensitizations at day 1 and day 8. *n* = 4. (**C**) Set-up of IL-4Rα blocking assay. Mice were injected with anti–IL-4Rα Ab as indicated in the diagram. Mice were sensitized with PCT at day 1 and day 8. Sera were collected at day 15, and Abs were tested using ELISA. (**D**) PN-specific IgE, PN-specific IgG1, and total IgE with IL-4Rα blocking after PCT. *n* = 3. *P < 0.05, **P < 0.01 by *t* test (**A**) or 1-way ANOVA (**B**–**D**). Data were representative of 1 (**C** and **D**) or 2 independent experiments (**A** and **B**).

Next, to better understand the IL-4 gene dosage effect we observed on the PN-specific IgE response, we tested the effect of the loss of 50% of IL-4 expression on IgE and IgG1 in our food allergy model using a different approach. We reasoned that, in *Il4*^+/–^ mice, where one *Il4* allele is normal and one allele is knocked out, IL-4 should be expressed at 50% lower levels than in WT mice. WT, *Il4*^+/–^, and *Il4*^–/–^ mice were thus sensitized for food allergy using PCT. Complete loss of *Il4* in *Il4*^–/–^ mice led to complete loss of the PN-specific IgE response and near complete loss of the IgG1 response (Figure 6A). In *Il4*^+/–^ mice, similar to *Il4*^–/–^ mice, PN-specific IgE was not detectable and there was very little total IgE, while these mice had similar levels of PN-specific IgG1 as WT mice (Figure 6A). Thus, unlike IgG1, IgE is extremely sensitive to IL-4 levels in this food allergy model. We further tested expression of IL-4 production by TFH and TFR cells in *Il4*^+/–^ mice using intracellular cytokine staining and found that, as expected, *Il4*^+/–^ TFH cells produced less IL-4 than WT cells and more IL-4 than KO cells (Figure 6B). TFH, TFR, and GC B cells were unchanged in *Il4*^–/–^ mice (Supplemental Figure 5, A–C). However, IgE^+^ GC B cells were significantly lower in *Il4*^–/–^ mice, as would be expected (Supplemental Figure 5D), suggesting that the level of IL-4 regulates the IgE response by inducing class switching to IgE. In a nonfood allergy model in which OVA-specific IgE is induced by injecting 2 doses of OVA plus Alum i.p., we also observed that OVA-specific IgE, but not IgG1, was severely impaired in *Il4*^+/–^ mice (Supplemental Figure 5E), a result consistent with previously published observations (10).

To gain a deeper insight into the role of IL-4 in the IgE response in the PCT food allergy system, we analyzed the timing of when IL-4 was critical for IgE production in our food allergy model. We therefore used an anti–IL-4Rα Ab to block the IL-4Rα signaling pathway at different time points during PCT sensitization. Specifically, in our 2-PCT sensitization model, we assessed the effects of blocking IL-4Rα after the first PCT sensitization, after the second PCT sensitization, and after both PCT sensitizations (Figure 6C). Compared with the control mice, PN-specific IgE failed to develop in mice in which IL-4 is blocked after the second PCT step, showing that IL-4 is essential for PN-specific IgE at the late priming stage in our model (Figure 6D). Early IL-4 may also be critical for PN-specific IgE, but we were not able to observe a statistically significant effect with only early blocking (Figure 6D). PN-specific IgG1 and total IgE were only modestly decreased by late-stage IL-4Rα blocking but were more strongly decreased by blocking at both early and late stages (Figure 6D). To exclude the possibility that IL-4Rα blocking impaired the TFH cells and the GC reaction, we examined TFH, TFR, and GC B cells in both spleen and mLN in these mice. We did not see significant differences in these populations (Supplemental Figure 5, F–H). These data show that, at the later stage of the PCT response, the development of IgE is extremely sensitive to circulating IL-4 levels.

### Endogenous TFR cells can produce IL-4 that contributes to Ag-specific IgE.

**Figure 7:**
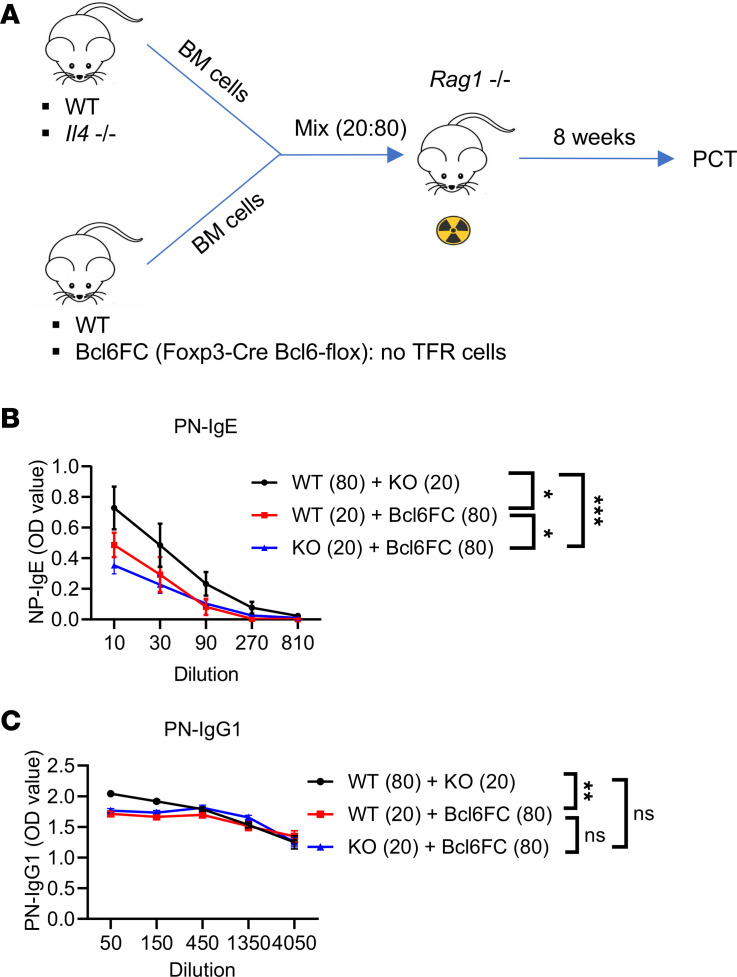
TFR cell–derived IL-4 can promote IgE in a BM chimera system. (**A**) Set-up of BM chimera (BMC) mice. BM cells from WT or *Il4*^–/–^ mice were mixed 20% + 80% with BM cells from Bcl6FC or WT mice. Mixed BM cells were injected (i.v.) into irradiated *Rag1*^–/–^ mice. Eight weeks later, BMC mice were sensitized with PCT. (**B**) PN-specific IgE in the 3 sets of BMC mice produced in **A** and sensitized with PCT at day 1 and day 8. Sera were collected, and Abs were tested at day 15. *n* = 4. (**C**) PN-specific IgG1 in BMC mice sensitized and assayed as in **B**. *P < 0.05, **P < 0.01, ***P < 0.001 by 2-way ANOVA. Data were representative of 2 independent experiments.

Here we decided to modify our BMC system to test the role of TFR-derived IL-4 more precisely than in our previous experiments. To avoid the severe *Il4* gene dosage issue of using 50% *Il4*^–/–^ cells as in Figures 5 and 6, we used 20% *Il4*^–/–^ BM so that the loss of IL-4 by TFH cells would be less severe. Thus, as shown in Figure 7, A and B, we produced 3 types of BMC mice. One set of control mice were 80% WT BM plus 20% *Il4*^–/–^ BM, one set of test mice were 80% Bcl6FC BM plus 20% WT BM, and the other set of test mice were 80% Bcl6FC BM plus 20% *Il4*^–/–^ BM. We expected in this last set of BMC mice that the 20% *Il4*^–/–^ BM would produce substantial numbers of IL-4–deficient TFR cells, whereas the Bcl6FC BM would not produce TFR cells. After PCT sensitization, robust levels of PN-specific IgE was produced in the 80% WT plus 20% *Il4*^–/–^ BMC mice (Figure 7B) showing that loss of 20% of IL-4–producing TFH/TFR cells did not ablate the PN-specific IgE response (in contrast to our previous BMC tests, in which 50% of the IL-4–producing TFH/TFR cells were lost; Figure 5). At the same time, as expected, BMC mice with 80% Bcl6FC BM plus 20% *Il4*^–/–^ BM showed a dramatically weaker PN-specific IgE response than the 80% WT BM plus 20% *Il4*^–/–^ BM mice (Figure 7B), showing the effect of loss of IL-4–producing TFR cells. A comparison of the IgE response for the 80% Bcl6FC BM plus 20% *Il4*^–/–^ BM mice versus the 80% Bcl6FC BM plus 20% WT BM mice showed that IL-4–producing TFR cells in the latter BMC set produced slightly but significantly higher PN-specific IgE (Figure 7B). As in earlier experiments, the IgG1 response was less affected by alterations in the TFR compartment (Figure 7C). Notably, a low level of PN-specific IgE was produced in the Bcl6FC + *Il4*^–/–^ BMC (Figure 7B), which indicates IL-4–independent effects of the TFR cells on the IgE response and may point to the effect of TFR-derived IL-10 (18). At the same time, the PN-specific IgE response in the WT + *Il4*^–/–^ BMC was significantly higher than the Bcl6FC + WT BMC. These data may indicate that WT TFR cells cannot fully compensate for loss of TFR cells in an 80% Bcl6FC BM setting, like the lack of full compensation we saw for TFH cells in BoyJ plus Bcl6 cKO BMC mice (Supplemental Figure 5C). Nonetheless, these data support the larger idea that Ag-specific IgE is highly sensitive to the levels of IL-4 secreted by both TFH and TFR cells and also show that TFR-derived IL-4 is important for the IgE response in the food allergy model. Overall, our data show that the IgE response, but not the IgG1 response, is extremely sensitive to even partial loss of IL-4 during the food allergy response.

## Discussion

In this study, we discovered that, in a mouse food allergy model, TFR cells unexpectedly produce levels of IL-4 very similar to the levels of IL-4 produced by TFH cells and that IL-4 derived from TFR cells can promote Ag-specific IgE responses both in vitro and in vivo.

TFR cells have been traditionally viewed as suppressor cells in the GC, but a previous study from our lab showed that TFR cells can play a helper role in the GC reaction and especially the IgE response (18). While we and others reported that TFR cells promote the IgE response through producing IL-10 (18, 37), our findings indicate that TFR cells also promote Ag-specific IgE by producing IL-4. This finding adds a new dimension to the role of TFR cells in the IgE response.

Although the absolute number of IL-4–producing TFR cells is < 15% of IL-4–producing TFH cells, these TFR cells still contribute to IgE response, which suggests either that the food allergy IgE response is highly sensitive to IL-4 levels produced by follicular T cells or that TFR-derived IL-4 plays an outsized role in the IgE response. Our results here with heterozygous mutant *Il4 (Il4*^+/–^) mice and TFH cell–deficient BMC mice show that a loss of 50% of IL-4 expression either globally or in the GC led to a severe loss of Ag-specific IgE. These data fit a model where IL-4 in the GC is limiting and, thus, even IL-4 produced by TFR cells is essential for the normal IgE response. Therefore, apart from TFR cells being a key source of IL-10 that can promote IgE in food allergy (18), TFR cells are also a key source of IL-4 that can promote IgE in food allergy.

While both IL-4 and IL-10 are important in this food allergy model, IL-4 is a primary regulator of IgE and may play a more dominant role in the IgE response than IL-10. In our data using TFR and Treg transfers into Bcl6FC mice and using TFR cell–deficient BMC mice, we saw that *Il4*^–/–^ TFR cells/Tregs can enhance the IgE response over baseline, though these cells consistently could not promote IgE as well as WT TFR cells/Tregs. One relevant question is whether IL-4–expressing TFR cells coproduce IL-10 or whether separate populations of TFR cells produce these 2 cytokines. To look at this, we analyzed intracellular IL-4 and IL-10 in TFR cells by flow cytometry in the food allergy response. Strikingly, we found that IL-4 and IL-10 were largely produced by separate populations of TFR cells, with only a minor population of TFR cells coexpressing IL-4 and IL-10 (Supplemental Figure 6A). A similar pattern was observed for TFH cells, although TFH cells produce lower levels of IL-10. These data indicate that different subsets of TFH and TFR cells produce these key IgE-driving cytokines, and future research will be needed to better understand these 2 subsets.

Our results with a mouse food allergy model contrast with other studies on allergic airway inflammation models showing that loss of TFR cells led to an enhanced IgE response (23, 36). Thus, TFR cells suppress the GC and IgE response in an allergic airway response, whereas in a food allergy model, TFR cells help or promote the GC and IgE response. These data imply that the immune microenvironment can greatly affect TFR cell function, but the mechanisms that control these very different TFR-mediated regulatory pathways are unknown. As part of the current study, we examined gene expression in TFR cells in the allergic model where TFR cells repress IgE. Strikingly, we found that the repressive TFR cells that develop in allergic airway disease also upregulate *Il4* gene expression like TFR cells in the food allergy model (Supplemental Figure 6B). Thus, expression of IL-4 alone cannot explain the difference between helper and suppressive TFR cells, and the suppressive phenotype likely relies on the levels of suppressive Treg factors expressed by these cells. Much more research is necessary to better understand the mechanisms of how TFR cells regulate IgE in different physiological contexts.

The process of TFR cells expressing high levels of IL-4 that can promote IgE responses has not been previously documented. An early characterization of TFR cells reported very little *Il4* mRNA expression in TFR cells after SRBC immunization, compared with *Il4* mRNA expression in TFH cells (21). A review of different experiments analyzing TFR cell gene expression has shown that *Il4* mRNA is not typically expressed in TFR cells except in the gut (38). Here we have shown that TFR cells produce substantial *Il4* mRNA and functional IL-4 protein in the context of a food allergy model that involves allergic sensitization via the gut. Here we observed that the upregulation of IL-4 in TFR cells appears to occur during the differentiation of pre-TFR cells into full TFR cells. Chatila and colleagues showed that IL-4–expressing Tregs develop in a food allergy model based on an activating mutation in the *Il4ra* gene and that the IL-4 expressed by these Tregs was critical for the IgE response (30). While TFR cells were not specifically examined in this study, it is possible that they were detecting TFR cell gene expression and function within the Foxp3^+^ T cell population. Even if this is not the case, the Chatila study provides clear evidence that Foxp3^+^ T cells develop into IL-4–expressing cells in food allergy and that this IL-4 can drive IgE responses (30).

We additionally wondered what factors were driving *Il4* gene expression in TFR cells. The transcriptional regulation of the *Il4* gene in T cells has been well characterized (35). Th2 cells and TFH cells are both major producers of IL-4, but curiously, transcriptional regulation of the *Il4* gene differs between TFH and Th2 cells. Unlike Th2 cells, *Il4* expression in TFH cells is dependent on the 3′ conserved noncoding sequence 2 (CNS2) in the *Il4* gene locus (39, 40). IgE responses typically develop in allergic responses that also involve the development of both IL-4–expressing Th2 cells and IL-4–expressing TFH cells (frequently referred to as TFH2 cells) (41), but IgE production in the food allergy model is completely dependent on TFH cells (18). TFH cells can develop in normal numbers in the absence of the Th2 activating factor Stat6 (42), and initial studies suggest that the IL-4 produced by TFH cells was independent of Stat6 and Gata3 (39). However, a more recent study suggests that Stat6 is required for the development of IL-4–expressing TFH cells and, moreover, that Stat6 cooperates with Stat3, Irf4, Batf, and Maf to activate IL-4 gene transcription in TFH cells (43). Our analysis of differentially expressed genes (DEGs) between naive TFR cells and PCT-primed TFR cells revealed increased expression of AP-1 family members *Fos, Jun*, *Junb*, and also *c-maf*, factors known to promote IL-4 expression in T cells (35). However, *Gata3* was decreased in PCT-primed TFR cells as *Il4* expression increased, suggesting that IL-4 production in TFR cells is regulated differently from IL-4 in Th2 cells, which is dependent on Gata3 function. Our data support the idea that a unique signaling pathway in TFR cells is induced in the food allergy response that can upregulate the expression of AP-1 and other *Il4*–promoting transcription factors, leading to high-level *Il4* expression. Much more work is required to characterize the precise signaling pathway and transcription factors that control *Il4* expression in TFR cells.

Critically, our data show that Ag-specific IgE is much more sensitive to IL-4 levels in the GC cells than IgG1, which can explain the role of TFR cells in the IgE but not the IgG1 response. We further observed that a loss of IL-4 in the GC by depleting 50% of TFH cells reduced Ag-specific IgE but not IgG1. Overall, our findings show that IL-4 produced by TFH and TFR cells differentially affects Ag-specific IgE versus IgG1. Differential regulation of Ag-specific IgE and IgG1 by overall IL-4 expression has been previously reported (10, 44), but the key role of TFH and TFR cells was not analyzed. The exact location and timing of the class switching to IgE remain unclear, but our data indicate that it occurs in the GC since we can detect an expansion of IgE^+^ B cells in the GC and since the process is dependent on both TFH and TFR cells (18, 31, 45). It remains unclear if, like TFH cells, IL-4 expression changes during TFR cell differentiation and localization (27, 29, 46). Despite their differences in origin and differentiation, our knowledge of TFR cells in GC still lags behind that of TFH cells (47, 48). Further research is needed to gain a deeper understanding of the induction, production, and function of IL-4 produced by TFR cells.

While our data indicate that the dose of IL-4–produced TFH cells is the primary contributor to Ag-specific IgE levels, we show here that TFR cells can contribute to the IgE response by producing IL-4. At the same time, TFR cells promote the IgE response in this model through other pathways, such as the production of IL-10 and the inhibition of the differentiation of TFH cells into a Th1-like cytotoxic state (18, 49).

One key question is whether TFR cells localize in the GC in similar or different places than TFH cells and how frequently IL-4–producing TFR cells interact with GC B cells. Preliminary analysis of TFR and TFH cells in the GC using immunofluorescence staining of histological sections revealed that TFR cells are rare in the GC (Supplemental Figure 7A) and will require a dedicated study to understand their localization and interactions with other cells in the GC more thoroughly.

TFR cells have recently become more complex with the discovery that TFH cells can upregulate Foxp3 expression and then appear as a subclass of TFR cells (50, 51). These Foxp3^+^ TFH cells express the same flow cytometry markers most often used to identify TFR cells (CD4^+^ Bcl6^+^, Foxp3^+^, PD-1^hi^, CXCR5^+^). TFR cells can, therefore, be seen as a mixture of Foxp3^+^ TFH cells as well as conventional Treg-derived TFR (cTFR) cells. A key characteristic of Foxp3^+^ TFH cells is that they carry T cell Ag receptors (TCRs) similar to TFH cells, in contrast to cTFR cells, which carry TCRs similar to Tregs (50, 51). Foxp3^+^ TFH cells have been identified both in humans and in mice (50, 51), but their function is poorly understood. Foxp3^+^ TFH cells may be part of a negative feedback mechanism for the GC response, but it’s not clear if they have other functions or if they are present in TFR cells in our food allergy model. Besides performing complex TCR-Seq analyses, good methods to discriminate Foxp3^+^ TFH cells from cTFR cells are not available in the mouse. Le Coz et al. showed that, in the human system, CD38 expression could be used to discriminate Treg-derived TFR cells from TFH-derived TFR cells (51). We tested CD38 expression on Tregs, TFR cells, and TFH cells in our food allergy model and found that Tregs have a high level of CD38 expression, and this level is increased on TFR cells (Supplemental Figure 7B). TFH cells have less CD38 than both Tregs and TFR cells. Thus, while CD38 may be expressed uniquely on Foxp3^+^ TFH cells in the mouse, much more work is needed to validate this marker.

In conclusion, our current study has markedly increased our understanding of TFR cells, demonstrating that, in food allergy, TFR cells can produce IL-4 and regulate IgE in a complementary manner to TFH cells. Furthermore, we show that TFR cell–derived IL-4 contributed to the availability of IL-4 in GC and that TFR-derived IL-4 can promote the generation of IgE^+^ B cells. We also have shown that IgE responses are more sensitive to the levels of IL-4 than previously thought and that care needs to be taken in interpreting data from BMCs where TFH cells and/or IL-4 is deficient. Our findings suggest that the development of food allergen–specific IgE can be blocked by drugs that inhibit IL-4 signaling only partially. Such drugs could be used to inhibit the development food allergy in the clinic.

## Methods

### Sex as a biological variable.

Mice of both sexes were used for this study. Female mice typically showed more robust production of IgE than males, but we observed similar results with both sexes of mice. Because of this sex variability, IgE data shown in 1 graph or data panel represents data from 1 sex, most often female.

### Mice.

All mutant mice were on a C57BL/6 background except for *Il4* reporter (4Get) mice. B6.129(Cg)-*Foxp3^tm4(YFP/icre)Ayr^*/J (Foxp3-YFP) mice, C.129-*Il4^tm1Lky^*/J (*Il4*/GFP-enhanced transcript; 4Get), and C57BL/6-*Il4^tm1Nnt^*/J mice (*Il4*^–/–^; *Il4*-KO) mice were obtained from The Jackson Laboratory. Foxp3-YFP-*Il4*^–/–^ mice were achieved by crossing Foxp3-YFP mice with *Il4*^–/–^ mice. Foxp3-Cre Bcl6-flox (Bcl6FC) and Cd4-Cre Bcl6-flox (Bcl6 cKO) mice were described previously (52, 53). IgE reporter mice Igh-7^tm1.2Cdca^ (Verigem) were obtained from Chris Allen (UCSF, San Francisco, California, USA) (54). Mouse littermate comparisons were used whenever possible. Control and experimental mouse cohorts were age and sex matched. Mice were bred under specific pathogen–free conditions at the laboratory animal facility of the Indiana University School of Medicine.

### Mice sensitization and immunizations.

For gut sensitizations, mice were deprived of food for 2 hours; then, each mouse was fed 300 μL 1.5% NaHCO_3_ water in using an i.g. method. Thirty minutes later, each mouse was given 1 mg PN (Greer Laboratories) together with 10 μg cholera toxin (MilliporeSigma) (PCT) according to the setting of experiments (55, 56). For OVA used as an Ag in the food allergy model, OVA was to replace PN. For OVA plus Alum immunizations, 100 μg of OVA were mixed with Alum (MilliporeSigma) and then injected i.p. into mice. For SRBC immunization, mice were i.p. injected with 1 × 10^9^ SRBC (Rockland Immunochemicals) and were sacrificed at the indicated day. For house dust mite (HDM) challenges, HDM (Greer Laboratories) was diluted with PBS. Mice were challenged intranasally with 25 μg HDM 3 times a week for 2 weeks. Mice were sacrificed on the indicated days, and the mLN, intranasal LNs, and spleen were harvested. Serum was also collected at the indicated time points.

### BMCs.

*Rag1*^–/–^ mice were sublethally irradiated (350 Gy). After 4 hours, BM cells from donor mice were mixed and then injected i.v. (5 × 10^6^ total/recipient) into the *Rag1*^–/–^ mice. In a 50:50 BMC, 2.5 × 10^6^ of each type of BM would be mixed prior to injection. The lymphoid compartment in the recipients was allowed to constitute for 2 months before sensitization or immunization.

### IL-4R blocking.

To block the IL-4Rα signaling pathway, 200 μg purified NA/NE rat anti–mouse IL-4Rα Ab (BD Pharmingen, clone mIL4R-M1 [RUO]) was injected (i.p.) into mice at day 2, 5, 9, and/or 12. PBS was used as a control. Mice were sensitized with PCT at day 1 and day 8.

### Flow cytometry.

Cell suspensions from mLNs were prepared and filtered through a 70 μm cell strainer (Thermo Fisher Scientific). Cells were washed and diluted in PBS with 1% FBS and were stained with Fc block (BioLegend) for 5 minutes, followed by surface staining for the indicated markers. The following Abs obtained from BioLegend were used for surface staining: anti-FOXP3 (clone MF-14), anti-CD4 (clone RM4-5), anti-CXCR5 (clone L138D7), anti-PD-1 (clone 29F.1A12), anti-B220 (clone RA3-6B2), anti-GL7 (clone GL7), and anti-CD38 (clone 90). For staining of IgE^+^ B cells, cells were preincubated with a high concentration of anti-IgE mAb (RME-1, BD Biosciences) to block surface IgE. The cells were washed and permeabilized with a Fixation/Permeabilization Kit (BD Pharmingen). Then anti-IgE Ab (RME-1, BioLegend) was used for intracellular IgE staining (54). For intracellular IL-4 staining, a Fixation/Permeabilization Kit (BD Pharmingen) was used following surface staining. Anti–IL-4 (11B11, BioLegend) Ab was used, with rat IgG1 κ as isotype control. All samples were acquired on an LSR2 flow cytometer (Becton Dickinson) and analyzed with FlowJo V10.6 (Tree Star Inc.).

### Intracellular cytokine staining.

CD4^+^ T cells were isolated from mLN and were cultured with a complete medium supplemented with 100 ng/mL PMA and 1 μg/mL ionomycin at 37°C with 4% CO_2_. After 1 hour, BD GolgiPlug (1:1,000 at final concentration) was added, and cells were incubated for another 3 hours. Cells were harvested and washed with ice-cold PBS. Then cells were used for cell surface staining and intracellular staining using Fixation/Permeabilization Kit (BD Pharmingen).

### ELISA.

For the measurement of Ag-specific IgE, 96-well Nunc-Immuno plates (MilliporeSigma) were coated with 5 μg/mL IgE Ab (clone LO-ME-3, Bio-Rad) in 0.1M carbonate buffer (pH 9.5) overnight at 4°C. Wells were blocked with 1% BSA for at least 1 hour at room temperature, and diluted serum was added and incubated at room temperature for 2 hours. For PN-specific IgE, PN was labeled with biotin (MilliporeSigma) and added into wells for 1 hour. For OVA-specific IgE, OVA was labeled with biotin (MilliporeSigma) and added into wells instead. Poly-HRP streptavidin (Pierce Endogen) was then added and incubated for 0.5 hours (1:5,000). For the measurement of PN-specific IgG1, 96-well Nunc-Immuno plates were coated with 5 μg/mL PN in 0.1M carbonate buffer (pH 9.5) overnight at 4°C. For the measurement of OVA-specific IgG1, plates were coated with OVA protein instead. Wells were then blocked with 1% BSA for at least 1 hour at room temperature, and diluted serum was added and incubated at room temperature for 2 hours. A biotin conjugated anti-mouse IgG1 (clone A85-1, BD Pharmingen) was used as secondary Ab (2 μg/mL) followed by adding avidin-HRP (Invitrogen) for 0.5 hours (1:2,000). For the measurement of total IgE, 96-well Nunc-Immuno plates were coated with 2 μg/mL anti–mouse IgE (clone R35-72, BD Pharmingen) overnight at 4°C. Wells were blocked with 1% BSA for at least 1 hour at room temperature, and diluted serum was added and incubated at room temperature for 2 hours. A biotin conjugated anti–mouse IgE (clone R35-118, BD Pharmingen) was used as secondary Ab (2 μg/mL) followed by adding avidin-HRP (Invitrogen) for 0.5 hours (1:2,000). After the incubation with HRP, TMB Substrate Reagent Set (BD Pharmingen) was added for the reaction development.

### qPCR.

The mRNA expression of genes was measured using TaqMan Fast Advanced Master Mix (Thermo Fisher Scientific) in QuantStudio 6 Flex Real-Time PCR System (Thermo Fisher Scientific). The probes for *Il4* (Mm00445259), *Jun* (Mm07296811), *Junb* (Mm04243546), *Fos* (Mm00487425), *Maf* (Mm01546091), *Yy1* (Mm01327906), and *Tubb5* (Mm00495806) were purchased from Thermo Fisher Scientific.

### In vitro coculture.

Cell culture set-ups were based on the protocol described by Clement et al. (22, 36). B cells were isolated from Verigem mice after PCT sensitizations using EasySep Mouse B Cell Isolation Kit (Stemcell Technologies). WT TFH and WT TFR cells were sorted from Foxp3-YFP mice after PCT sensitizations. *Il4*^–/–^ TFR cells (KO-TFR) were sorted from Foxp3-YFP-*Il4*^–/–^ mice after PCT sensitizations. B cells (50,000 cells) were cultured with/without TFH (30,000 cells) and/or TFR (15,000 cells) cells in a complete medium. Peanut protein was added (20 μg/mL) into the coculture. Four days later, IgE^+^ B cells were analyzed using flow cytometry.

### RNA-Seq and analysis.

Mice were sensitized with PCT twice; then, TFR cells were sorted from mesenteric LNs. TFR cells from naive mice were also sorted as controls. Mesenteric LNs from around 4–5 mice were combined to 1 sample. Around 20,000–40,000 cells were sorted for 1 sample. RNA-Seq was performed by the Indiana University School of Medicine Center for Medical Genomics. Uniquely mapped sequencing reads were assigned to mm10 refGene genes. Quality control of sequencing and mapping results was summarized using MultiQC. Genes with read count per million (CPM) < 0.5 in more than 4 of the samples were removed. The data were normalized using trimmed mean of M (TMM) values method. Differential expression analysis was performed using edgeR. FDR was computed from *P* values using the Benjamini-Hochberg procedure. DEGs were determined if their *P* values were less than 0.05 after multiple-test correction with FDR adjustment and the amplitude of fold changes (FCs) were larger than 1.8. The RNA-Seq data can be accessed via the NCBI GEO database (accession nos. GSE226612, GSE202713). Heatmaps were created using pheatmap package with clustering rows in R (4.3.1).

### Immunofluorescence staining and microscopy.

Foxp3-YFP mice were sensitized with PCT i.g. by the normal protocol. At day 15, mLN were taken and embedded with OCT (TissueTek) and frozen on dry ice. Cryostat sections (7 μm) were prepared on slides and were then fixed with PBS containing 2% PFA before being blocked and permeabilized with buffer (PBS with 1% BSA, 0.3% Triton X-100, and 5% mouse and rat serum; in-house) prior to staining with the Ab cocktail: BV421-conjugated anti-CD4 (BioLegend, 100437), Al488-conjugated anti-FOXP3 (BioLegend, 320012), PE-conjugated anti-BCL6 (BioLegend, 358504), and Al647-conjugated anti-CR1/2 (BioLegend, 123424). Images were captured with an DMi8 fluorescence microscope from Lecia company with Leica LAS X software.

### Statistics.

All data analysis was performed using GraphPad Prism software (GraphPad Software). Data are shown as the mean ± SEM. Unless otherwise stated, 2-tailed Student’s *t* test or 1-way ANOVA with Tukey’s post hoc analysis was used. All ELISAs were analyzed using 2-way ANOVA. Significant differences (*P* < 0.05) and some nonsignificant differences are indicated in the figures. Further statistical details of experiments can be found in figure legends. The investigators were not blinded to the analyses.

### Study approval.

All experiments and handling of animals were conducted according to protocols approved by the IACUC of the Indiana University School of Medicine.

### Data availability.

Underlying data for graphs are available in the Supporting Data Values file. Bulk RNA-Seq data are available from NCBI (GEO, accession no. GSE226612). Any data or further information is available from the corresponding author on request.

## Author contributions

QC helped design the study, performed several of the experiments, analyzed results, prepared figures, and wrote the manuscript. AMA performed several of the experiments, analyzed results, prepared figures, and helped write the manuscript. WL and XY performed immunofluorescence analysis of GC sections. ALD helped design the study, analyzed the results, and wrote the manuscript. QC was given first listing, as he initiated the project.

## Supplementary Material

Supplemental data

Supporting data values

## Version 1 10/08/2024

Electronic publication
