# Supplementary material for: T follicular regulatory cells in food allergy promote IgE via IL-4
Source: JCI Insight. 2024 Oct 8;9(19):e171241. doi: 10.1172/jci.insight.171241 (PMC11466180; doi:10.1172/jci.insight.171241)
Supplement: Supplemental data [file jciinsight-9-171241-s110.pdf]

**Supplemental information**

**Supplemental Figure 1**. (A) Gating strategy used in flow cytometry to detect TFH and TFR cells after PCT sensitization. Foxp3 expression was assessed by intracellular staining. (B) Absolute numbers of *Il4*-$GFP^+$ TFH versus TFR cells in mesenteric lymph node (mLN), from gating in A with *Il4*-$GFP^+$ mice and calculation of cell numbers from total mLN cells. n = 5. **** P < 0.0001 by Students T-test. Data were representative of two independent experiments.

**Supplemental Figure 2.** (A) Gating strategy used in flow cytometry to measure *Il4*-GFP-expressing Treg cell populations in 4Get mice, using the same gating approach as in Sup. Fig. 1. $CD4^+Foxp3^+$ Treg cells were divided into four groups based on CXCR5 and PD-1 expression. *Il4*-$GFP^+$ cells were measured in these four sub-populations. (B) Percentages of *Il4*-$GFP^+$ cells in pre-TFR ($CXCR5^+PD\text{-}1^-$) and TFR ($CXCR5^{high}PD\text{-}1^{high}$) populations in naive mice, mice with SRBC immunizations, and mice sensitized with PCT. (C) *Il4* mRNA expression in TFH cells and TFR cells after PCT sensitization. Mice were sensitized either with PCT at day 1 and cells isolated by FACS at day 8 for RNA preparation or PCT at day 1 and day 8, and cells isolated by FACS at day 12 for RNA preparation for RNA preparation. n =4. (D) Percentages of *Il4*-$GFP^+$ cells in TFR ($CXCR5^{high}PD\text{-}1^{high}$) cells in different lymphoid organs from naive mice. “other LNs” is inguinal, popliteal and sublingual LNs.  * P < 0.05, ** P < 0.01, *** P < 0.001, **** P < 0.0001, ns not significant by ANOVA. (A-B) n = 8. (C-D) n=4.

**Supplemental Figure 3.** Heatmap of *Il4* regulating genes, comparing bulk RNAseq of TFH and TFR cells from day 1 and day 8 PCT sensitized mice.

**Supplemental Figure 4.** (A) Bone marrow chimera (BMC) design and experimental flow, where CD45 congenically marked mice could distinguish between the two types of input bone marrow. Irradiated Rag1-KO mice received equal amount of BM cells from BoyJ (CD45.1) and Bcl6-flox or Bcl6 cKO mice. Eight weeks later, the mice were primed for peanut allergy with PCT and donor derived TFH and TFR cells percentages were measured by flow cytometry. (B) The contribution of each type of donor to the TFH and TFR cell compartments for the two types of BMC. (C) Percentages of TFH and TFR cells in the two types of BMC. (D) PN-specific IgE levels in the two types of BMC at day 15 of the PCT response.

**Supplemental Figure 5.** (A-D) WT mice and Il4 -/- mice were sensitized with PCT. The percent of TFH cells (A), TFR cells (B), GC B cells (C), and $IgE^{+}$ GC B cells (D) were analyzed in the spleen and mLN using flow cytometry. (E) Ab response in mice immunized with OVA plus Alum. OVA-specific IgE, OVA-specific IgG1, and total IgE in WT, *Il4* +/-, and *Il4* -/- mice after immunization with OVA plus Alum. Mice were immunized with OVA plus Alum at day 1 and day 8. Sera were collected at day 15, and Abs were tested using ELISA. n = 8. Data were representative of two independent experiments. (F-H) TFH, TFR, and GC B cells were unaffected by anti-IL-4Rα blocking or Il4 gene knockout. Mice were injected with anti-IL-4Rα Abs. Mice were also sensitized with PCT. The percent and cell number of TFH cells (F), TFR cells (G), and GC B cells (H) in the spleen and mLN were analyzed using flow cytometry. n = 7–9. n = 5. * P < 0.05, ** P < 0.01, *** P < 0.001, **** P < 0.0001, ns not significant by t-test (A-H). Data were representative of two or three independent experiments.

**Supplemental Figure 6.** (A) The expression of IL-4 and IL-10 in TFH and TFR cells after PCT sensitizations. The percentages of IL-4$^-$IL-10$^-$, IL-4$^+$IL-10$^-$, IL-4$^-$IL-10$^+$, and IL-4$^+$IL-10$^+$ TFH and TFR cells were measured via flow cytometry after cell fixation, permeabilization and intracellular cytokine staining. (B) Heatmap of Th2-related genes in TFR cells from HDM allergic airway primed mice. TFR cells were sorted from the mediastinal LNs of naïve mice and mice after intranasal HDM challenges. Total RNA was extracted and subjected to bulk RNA-seq and analysis. The heatmap represents log2FPKM values.

**Supplemental Figure 7.** (A) Image shows frozen section of mLN at 7 days after PCT sensitization after immunofluorescent staining. Red – Bcl6, Blue – CD21/35, Green – Foxp3. The dark zone of GC stains intensely for Red Bcl6+ B cells, while the GC light zone will have blue CD21/CD35+ FDCs. TFH cells should be Red with a white border and TFR cells should be red/green/yellow with a white border. Yellow arrows show likely TFH cells. Red and green arrow shows likely TFR cell. GCs found in the mLN after PCT priming were relatively small when compared to splenic GCs after i.p. immunization. (B) CD38 expression on Treg cells, TFR cells, and TFH cells after PCT sensitization, assayed by flow cytometry. ** $P < 0.01$, *** $P < 0.001$, ns not significant by one-way ANOVA. n = 3.

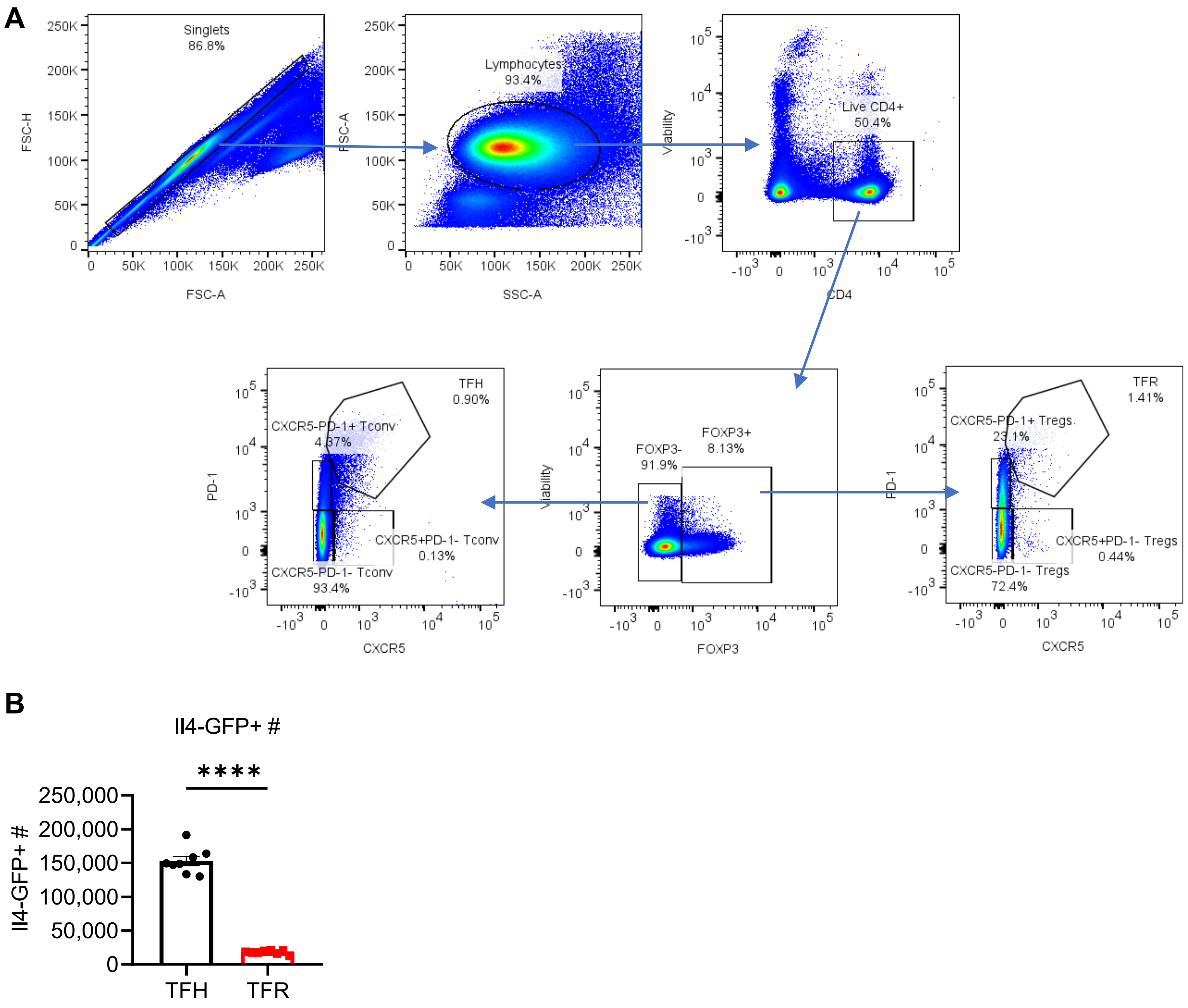
A
Singlets
86.8%
FSC-H
FSC-A
Lymphocytes
93.4%
SSC-A
Live CD4+
50.4%
Viability
CD4
TFH
0.90%
CXCR5-PD-1+ Tconv
4.37%
CXCR5+PD-1- Tconv
0.13%
CXCR5-PD-1- Tconv
93.4%
PD-1
CXCR5
FOXP3+
8.13%
FOXP3-
91.9%
FOXP3
TFR
1.41%
CXCR5-PD-1+ Tregs
23.1%
CXCR5+PD-1- Tregs
0.44%
CXCR5-PD-1- Tregs
72.4%
B
Il4-GFP+ #
****
250,000
200,000
150,000
100,000
50,000
0
TFH
TFR

Sup. Fig. 1

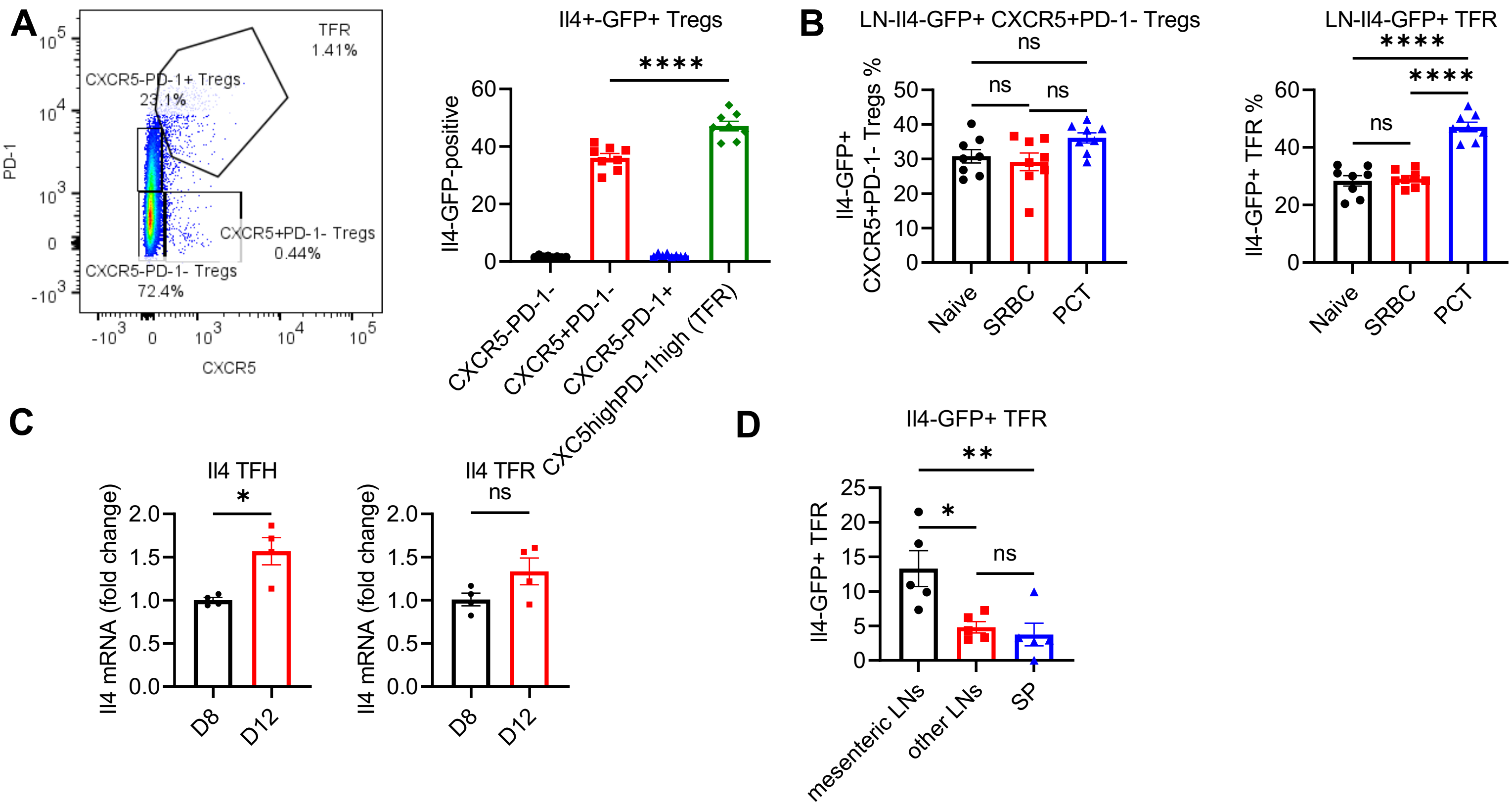

A
TFR
1.41%
CXCR5-PD-1+ Tregs
23.1%
CXCR5+PD-1- Tregs
0.44%
CXCR5-PD-1- Tregs
72.4%
PD-1
CXCR5
Il4+-GFP+ Tregs
Il4-GFP-positive
****
CXCR5-PD-1-
CXCR5+PD-1-
CXCR5-PD-1+
CXC5highPD-1high (TFR)
B
LN-Il4-GFP+ CXCR5+PD-1- Tregs
Il4-GFP+ CXCR5+PD-1- Tregs %
ns
Naive
SRBC
PCT
LN-Il4-GFP+ TFR
Il4-GFP+ TFR %
****
C
Il4 TFH
Il4 TFR
Il4 mRNA (fold change)
*
ns
D8
D12
D
Il4-GFP+ TFR
Il4-GFP+ TFR
**
*
ns
mesenteric LNs
other LNs
SP


Sup. Fig. 2

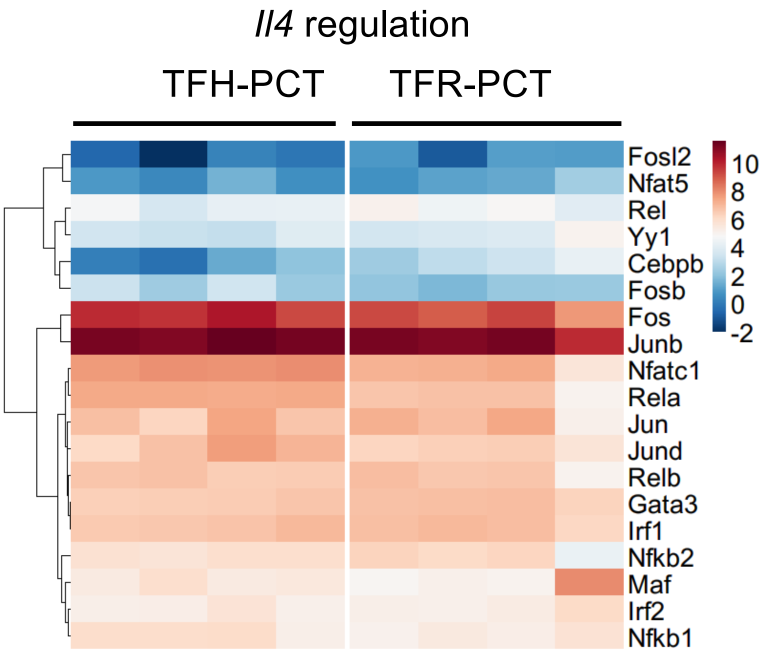
*Il4* regulation
TFH-PCT
TFR-PCT
Fosl2
Nfat5
Rel
Yy1
Cebpb
Fosb
Fos
Junb
Nfatc1
Rela
Jun
Jund
Relb
Gata3
Irf1
Nfkb2
Maf
Irf2
Nfkb1
10
8
6
4
2
0
-2

Sup. Fig. 3

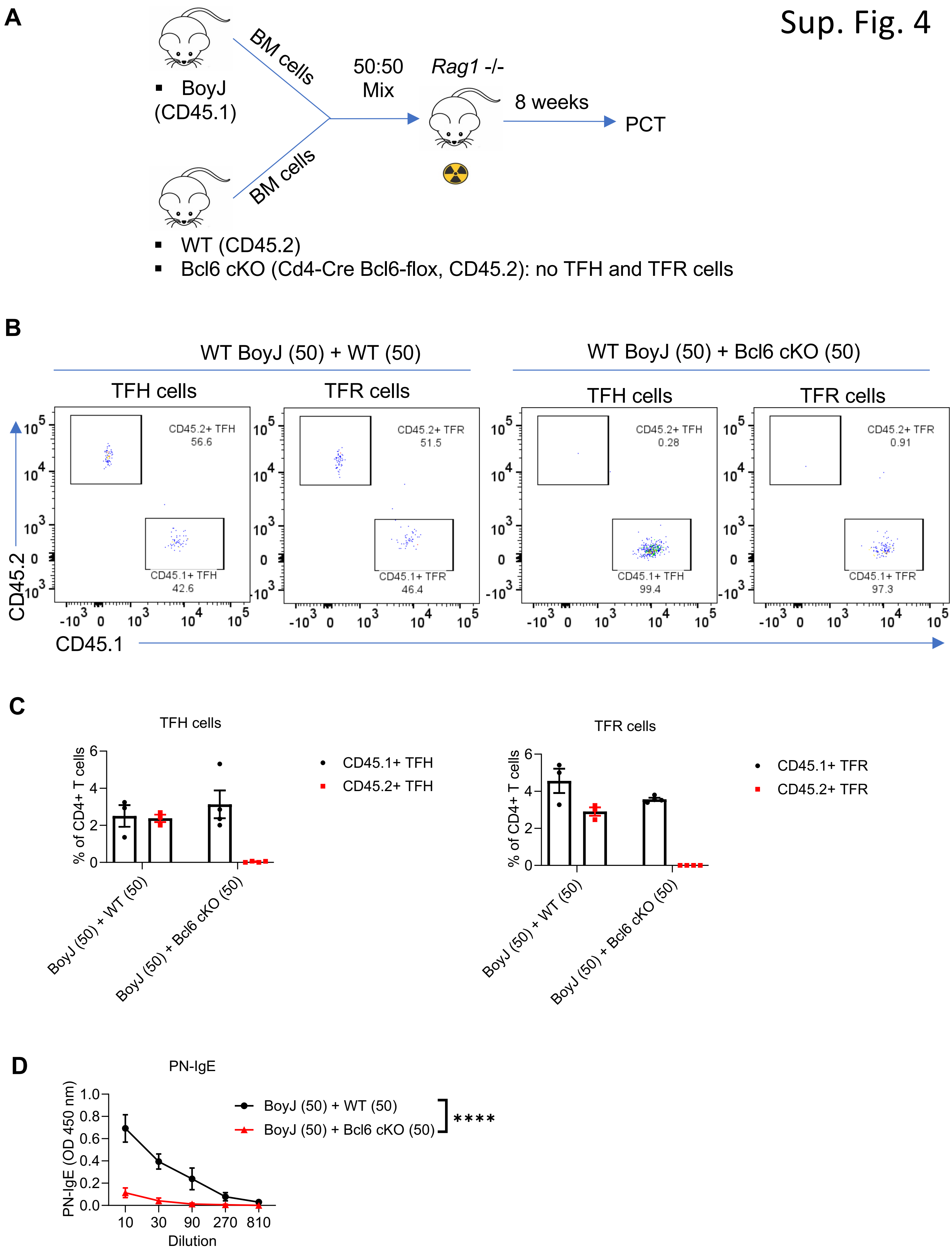

Sup. Fig. 4
A
BoyJ (CD45.1)
BM cells
50:50 Mix
Rag1 -/-
8 weeks
PCT
WT (CD45.2)
Bcl6 cKO (Cd4-Cre Bcl6-flox, CD45.2): no TFH and TFR cells
B
WT BoyJ (50) + WT (50)
WT BoyJ (50) + Bcl6 cKO (50)
TFH cells
TFR cells
CD45.2+ TFH 56.6
CD45.1+ TFH 42.6
CD45.2+ TFR 51.5
CD45.1+ TFR 46.4
CD45.2+ TFH 0.28
CD45.1+ TFH 99.4
CD45.2+ TFR 0.91
CD45.1+ TFR 97.3
CD45.2
CD45.1
C
TFH cells
TFR cells
% of CD4+ T cells
CD45.1+ TFH
CD45.2+ TFH
CD45.1+ TFR
CD45.2+ TFR
BoyJ (50) + WT (50)
BoyJ (50) + Bcl6 cKO (50)
D
PN-IgE
PN-IgE (OD 450 nm)
BoyJ (50) + WT (50)
BoyJ (50) + Bcl6 cKO (50)
****
Dilution
10 30 90 270 810

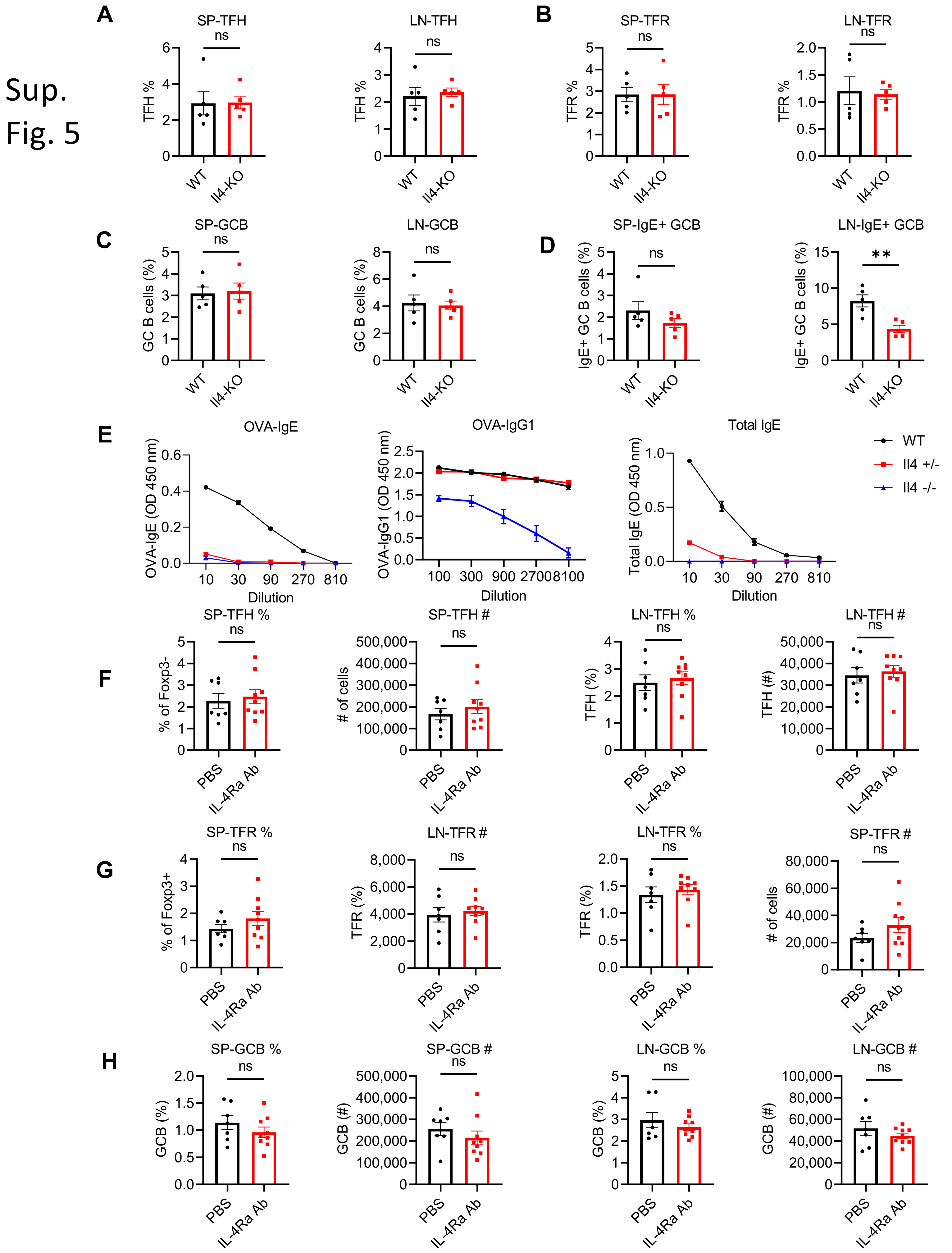
Sup. Fig. 5
A
SP-TFH
ns
TFH %
WT
Il4-KO
LN-TFH
B
SP-TFR
TFR %
LN-TFR
C
SP-GCB
GC B cells (%)
LN-GCB
D
SP-IgE+ GCB
IgE+ GC B cells (%)
LN-IgE+ GCB
**
E
OVA-IgE
OVA-IgE (OD 450 nm)
Dilution
OVA-IgG1
OVA-IgG1 (OD 450 nm)
Total IgE
Total IgE (OD 450 nm)
WT
Il4 +/-
Il4 -/-
F
SP-TFH %
% of Foxp3-
PBS
IL-4Ra Ab
SP-TFH #
# of cells
LN-TFH %
TFH (%)
LN-TFH #
TFH (#)
G
SP-TFR %
% of Foxp3+
LN-TFR #
TFR (%)
LN-TFR %
SP-TFR #
H
SP-GCB %
GCB (%)
SP-GCB #
GCB (#)
LN-GCB %
LN-GCB #

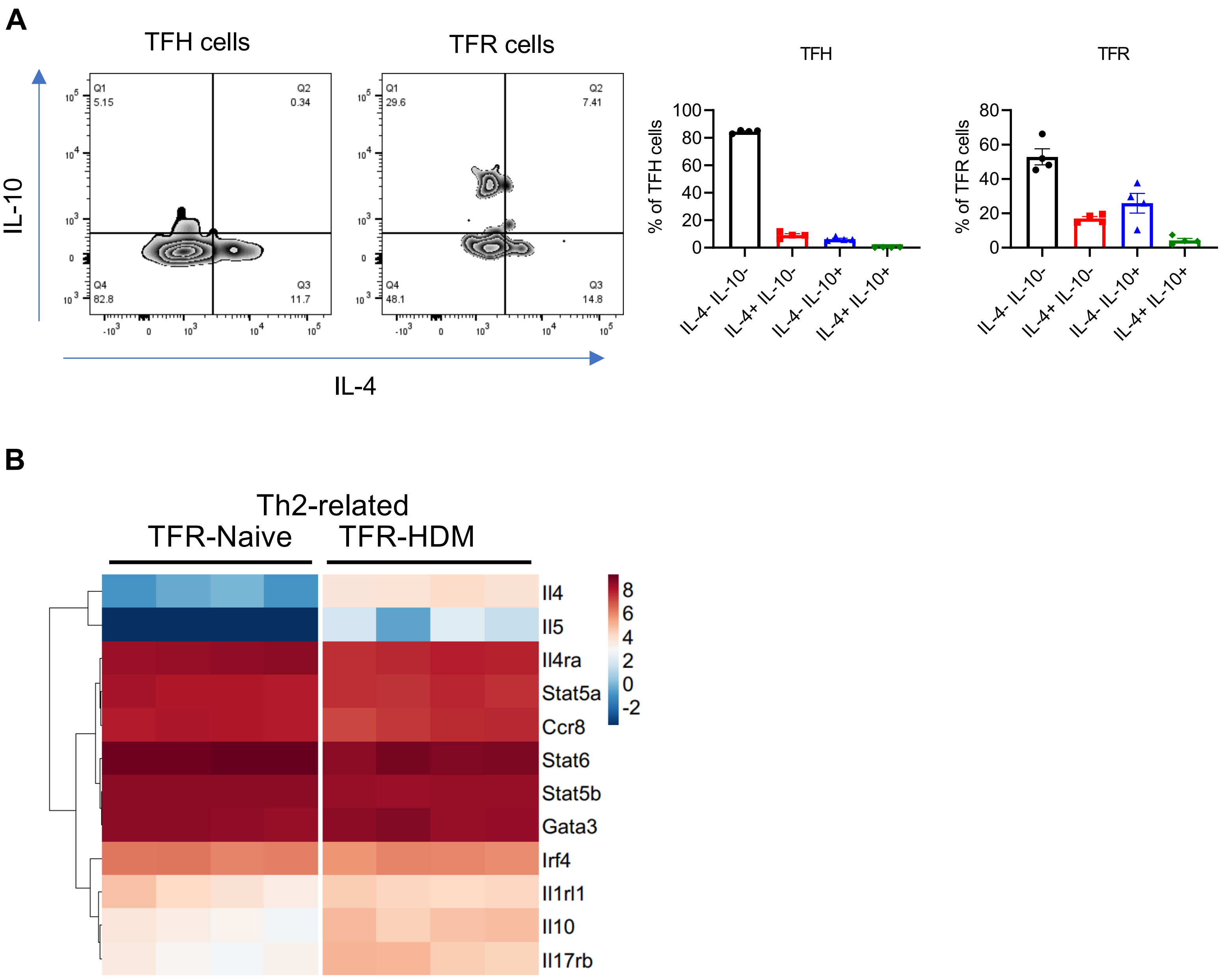
A
TFH cells
TFR cells
IL-10
IL-4
Q1
5.15
Q2
0.34
Q4
82.8
Q3
11.7
Q1
29.6
Q2
7.41
Q4
48.1
Q3
14.8
TFH
% of TFH cells
TFR
% of TFR cells
IL-4- IL-10-
IL-4+ IL-10-
IL-4- IL-10+
IL-4+ IL-10+
B
Th2-related
TFR-Naive
TFR-HDM
Il4
Il5
Il4ra
Stat5a
Ccr8
Stat6
Stat5b
Gata3
Irf4
Il1rl1
Il10
Il17rb

Sup. Fig. 6

**A**

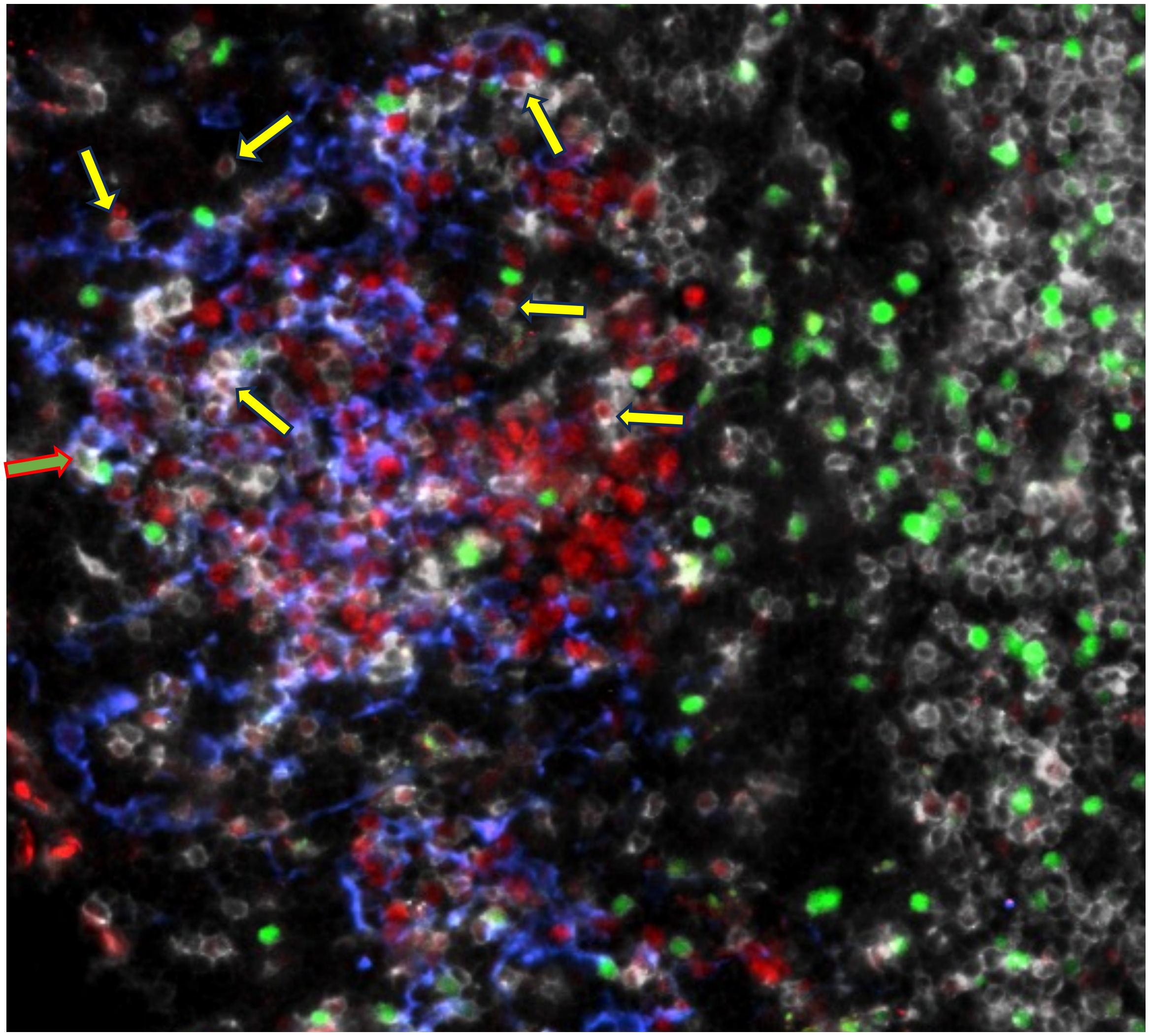

**B**

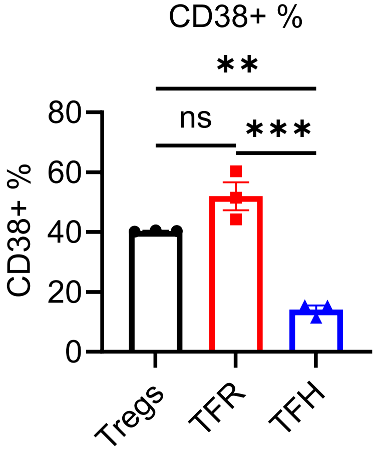


Sup. Fig. 7
